# Metabolic versatility of *Caldarchaeales* from geothermal features of Hawai’i and Chile as revealed by five metagenome-assembled genomes

**DOI:** 10.3389/fmicb.2023.1216591

**Published:** 2023-09-20

**Authors:** Manolya Gul Balbay, Maximillian D. Shlafstein, Charles Cockell, Sherry L. Cady, Rebecca D. Prescott, Darlene S. S. Lim, Patrick S. G. Chain, Stuart P. Donachie, Alan W. Decho, Jimmy H. Saw

**Affiliations:** ^1^Department of Biological Sciences, The George Washington University, Washington, DC, United States; ^2^UK Centre for Astrobiology, University of Edinburgh, Edinburgh, United Kingdom; ^3^Department of Geology, Portland State University, Portland, OR, United States; ^4^School of Life Sciences, University of Hawai’i at Mānoa, Honolulu, HI, United States; ^5^Department of Environmental Health Sciences, University of South Carolina, Columbia, SC, United States; ^6^Department of Biology, University of Mississippi, Oxford, MS, United States; ^7^NASA Ames Research Center, Moffett Field, CA, United States; ^8^Los Alamos National Laboratory, Los Alamos, NM, United States

**Keywords:** Aigarchaeota, *Caldarchaeales*, extremophiles, fumaroles, hot springs, metabolism

## Abstract

Members of the archaeal order *Caldarchaeales* (previously the phylum Aigarchaeota) are poorly sampled and are represented in public databases by relatively few genomes. Additional representative genomes will help resolve their placement among all known members of *Archaea* and provide insights into their roles in the environment. In this study, we analyzed 16S rRNA gene amplicons belonging to the *Caldarchaeales* that are available in public databases, which demonstrated that archaea of the order *Caldarchaeales* are diverse, widespread, and most abundant in geothermal habitats. We also constructed five metagenome-assembled genomes (MAGs) of *Caldarchaeales* from two geothermal features to investigate their metabolic potential and phylogenomic position in the domain *Archaea*. Two of the MAGs were assembled from microbial community DNA extracted from fumarolic lava rocks from Mauna Ulu, Hawai‘i, and three were assembled from DNA obtained from hot spring sinters from the El Tatio geothermal field in Chile. MAGs from Hawai‘i are high quality bins with completeness >95% and contamination <1%, and one likely belongs to a novel species in a new genus recently discovered at a submarine volcano off New Zealand. MAGs from Chile have lower completeness levels ranging from 27 to 70%. Gene content of the MAGs revealed that these members of *Caldarchaeales* are likely metabolically versatile and exhibit the potential for both chemoorganotrophic and chemolithotrophic lifestyles. The wide array of metabolic capabilities exhibited by these members of *Caldarchaeales* might help them thrive under diverse harsh environmental conditions. All the MAGs except one from Chile harbor putative prophage regions encoding several auxiliary metabolic genes (AMGs) that may confer a fitness advantage on their *Caldarchaeales* hosts by increasing their metabolic potential and make them better adapted to new environmental conditions. Phylogenomic analysis of the five MAGs and over 3,000 representative archaeal genomes showed the order *Caldarchaeales* forms a monophyletic group that is sister to the clade comprising the orders *Geothermarchaeales* (previously *Candidatus* Geothermarchaeota), *Conexivisphaerales* and *Nitrososphaerales* (formerly known as Thaumarchaeota), supporting the status of *Caldarchaeales* members as a clade distinct from the Thaumarchaeota.

## Introduction

The archaeal candidate phylum Aigarchaeota belongs to the TACK superphylum, which initially consisted of the Thaumarchaeota, Aigarchaeota, Crenarchaeota and Korarchaeota phyla (Guy and Ettema, 2011; Adam et al., 2017). The TACK superphylum now comprises several additional phyla discovered since 2011, including Bathyarchaeota (Meng et al., 2014), Verstraetearchaeota (Vanwonterghem et al., 2016), Geothermarchaeota (Jungbluth et al., 2017), and Nezhaarchaeota (Wang et al., 2019). Representatives of the Aigarchaeota, originally designated HWCG-I (Hot Water Crenarchaeotic Group I) were first discovered in a microbial mat collected from a moderately acidic geothermal stream in a subsurface gold mine (Nunoura et al., 2011). A genome fragment of HWCG-I in a fosmid clone from the metagenomic library of a microbial mat community thriving in hydrothermal fluid at the gold mine was sequenced and annotated (Nunoura et al., 2005). Intriguingly, one of the genes identified in the genome encodes a potential aerobic-type carbon monoxide dehydrogenase, which indicates that HWCG-I might be capable of chemolithotrophic growth using carbon monoxide as an electron donor and molecular oxygen as an electron acceptor, and that HWCG-I might be a facultative or obligate aerobe (Nunoura et al., 2005). Later, a composite circular genome sequence of an HWCG-I member, *Candidatus* “Caldiarchaeum subterraneum” (hereafter abbreviated as *Ca. C. subterraneum*), was assembled from the metagenomic library (Nunoura et al., 2011). Characterization of the genome of *Ca. C. subterraneum* revealed that the organism may perform hydrogenotrophy, aerobic carbon monoxide oxidation, aerobic respiration, and anaerobic respiration via nitrate or nitrite reduction (Nunoura et al., 2011). These archaea may also perform carbon fixation using the dicarboxylate/4-hydroxybutyrate pathway, though it lacks a key marker enzyme (4-hydroxybutyryl-CoA dehydratase) for this carbon fixation pathway.

Even though the presence of NiFe hydrogenases in the genome of *Ca. C. subterraneum* led to the suggestion that this organism is capable of hydrogenotrophic lifestyle (Nunoura et al., 2011), the hydrogenases in *Ca. C. subterraneum* were found to belong to Group 3B and Group 4 NiFe hydrogenases, which are involved in the regulation of redox homeostasis rather than hydrogenotrophy (Vignais, 2008; Hedlund et al., 2015). Nunoura et al. (2011) also identified genes previously found only in eukaryotes, such as a potential ubiquitin protein modification system in *Ca. C. subterraneum*. The genome of HWCG-I was proposed to belong to a novel candidate archaeal phylum, “Aigarchaeota,” since the genome of *Ca. C. subterraneum* possessed unique characteristics that distinguish it from the previously described genomes of the phyla Crenarchaeota, Euryarchaeota, Thaumarchaeota, and Korarchaeota (Nunoura et al., 2011).

A single-cell genomics study, which generated 14 Aigarchaeota single-cell amplified genomes (SAGs) from sediments (~75°C–85°C) in Great Boiling Spring, Nevada (Rinke et al., 2013) revealed that they constitute five different species-level groups, and that each of those species-level groups is distinct from *Ca. C. subterraneum* (Rinke et al., 2013; Hedlund et al., 2014). Characterization of the SAGs revealed the presence of a large subunit of the ribulose-1,5-bisphosphate carboxylase oxygenase (RuBisCO) enzyme in a SAG, indicating that the Aigarchaeota may have the potential to fix carbon through the Calvin-Benson-Bassham cycle (CBB; Rinke et al., 2013). Some of the SAGs also have genomic potential for aerobic respiration, anaerobic respiration via dissimilatory sulfite reduction and reduction of nitrous oxide, and heterotrophic utilization of proteins and sugars (Rinke et al., 2013; Hedlund et al., 2014, 2015). In 2016, a novel member of Aigarchaeota, *Candidatus* "Calditenuis aerorheumens” was described based on both metagenome and metatranscriptome analyses of the hyperthermophilic, filamentous “pink streamer” communities in the Octopus Spring, Yellowstone National Park (Beam et al., 2016). It was reported that this archaeon could be an aerobic chemoorganotroph with autotrophic potential and is likely to able to utilize an array of organic carbon substrates, including acetate, fatty acids, amino acids and sugars, and thus may be important in cycling dissolved organic carbon (Beam et al., 2016).

Phylogenetic, phylogenomic, and comparative genomic studies have consistently unraveled a deep relationship between Thaumarchaeaota, Aigarchaeota, Crenarchaeota, and Korarchaeota in the “TACK superphylum” (Guy and Ettema, 2011; Wolf et al., 2012; Rinke et al., 2013; Hedlund et al., 2015; Petitjean et al., 2015), yet it remains controversial whether Aigarchaeota represents an independent phylum or a subclade of the phylum Thaumarchaeota (Brochier-Armanet et al., 2011; Guy and Ettema, 2011; Gribaldo and Brochier-Armanet, 2012; Spang et al., 2013; Hedlund et al., 2015). Distinct ecophysiologies of these two lineages were evident from an analysis of Aigarchaeota genome bins recovered from hot spring sediments in Tengchong, China, which revealed a strict or facultatively anaerobic lifestyle, sulfide oxidation for energy conservation, and substantial gene loss from their early ancestors (Hua et al., 2018). They also displayed diversity both in metabolic pathways and ecological roles, indicating functional partitioning and ecological divergence within a single geothermal region (Hua et al., 2018). Evolutionary genomic analyses of Aigarchaeota and its sister lineage Thaumarchaeota suggested that both phyla originated in thermal environments, sharing a large proportion of gene families with their thermophilic last common ancestor and later migration and adaptation of Thaumarchaeota to a wide range of non-thermal habitats led to the functional differentiation between these two groups of *Archaea* (Hua et al., 2018). Phylogenetic and phylogenomic analyses of 14 Aigarchaeota and 80 Thaumarchaeota genomes in the same study provided evidence that they are different phyla (Hua et al., 2018). However, a more recent effort to unify taxonomic classification of prokaryotes led to the phylum Aigarchaeota being demoted and being reclassified and renamed as the order *Candidatus* Caldarchaeales in the Genome Taxonomy Database (GTDB; Rinke et al., 2021), and we will refer to it as the order *Caldarchaeales* without the Candidatus designation throughout the text for brevity.

Here, we report previously uncharacterized metagenome-assembled genomes (MAGs) of members of *Caldarchaeales* obtained from geothermal habitats from Hawai‘i and Chile and showed that they have genomic potential for metabolic versatility. We also showed that the addition of these previously uncharacterized MAGs led to improved phylogenomic resolution of the order *Caldarchaeales* and increased the representation of poorly sampled members of this enigmatic archaeal group.

## Materials and methods

### Sample collection and processing

Fieldwork, sampling, and DNA extraction of lava rock samples from active fumaroles in Mauna Ulu volcanic area in Hawai‘i were previously reported, and the MAGs in this study originated from sample numbered 86B (Cockell et al., 2019; Hughes et al., 2019). Biofilm samples from two hot springs in El Tatio geothermal field in Chile were collected during a field trip in August 2018. The MAGs reported here came from samples collected at two different hot spring features: Cacao East 5 (abbreviated as CE5; GPS coordinates of −22.350367, −68.008050) and Poppy (GPS coordinates of −22.333478, −68.013011) pools. A field photo of Poppy pool is shown in Supplementary Figure S1 and that of CE5 has been reported previously (Megevand et al., 2022). At the time of collection, the main CE5 pool had a temperature of 83.4°C and pH of 7.2, whereas the main Poppy pool had a temperature of 78.3°C and pH of 6.85. Sample material for DNA extraction from Poppy consisted of a brown-colored biofilm scraped from a piece of hot spring sinter that was fractured with sterile tweezers off the rim of the actively splashing hot spring. Sample material for DNA extraction from the more quiescent CE5 pool was collected with a sterile tweezer as a vertical section of a completely submerged hot spring mat located just below the water line at the pool’s edge. After collection, the hot spring sinter and mat samples were placed into sterile Qorpak jars (Berlin Packaging, Clinton, PA), and transported within a few hours to a hotel where they were stored at ~4°C overnight. The sample jars were then transported on ice to the PNNL laboratory where they were stored at −80°C until they were shipped on blue ice to the University of Hawai‘i laboratory, where they remained frozen until they were processed for DNA extraction. DNA was extracted from 0.5 g of each sample using the Qiagen DNeasy PowerSoil DNA kit and quantified with a Qubit instrument.

### Sequencing

Illumina libraries of samples from Chile were prepared using NEBNext Ultra DNA II Library Preparation Kit (New England Biolabs, Cat. #E7645L). Sequencing libraries were generated from sample material that had input DNA amount varying between 5 and 100 ng. The DNA was fragmented with a Covaris E220, the ends made blunt, and adapters and indexes added to blunt ends for sequencing on an Illumina sequencer. Illumina libraries were eluted in DNA Elution Buffer (Zymo Research, Cat. #D3004-4-10), and concentrations of the libraries were obtained using the Qubit dsDNA HS Assay (ThermoFisher Scientific, Cat. #Q32854). The average size of the DNA in the library was determined by the Agilent High Sensitivity DNA Kit (Agilent, Cat. #5067–4,626). Libraries were quantified with the Library Quantification Kit – Illumina/Universal Kit (KAPA Biosystems, KK4824), and sequenced using a NextSeq v2.5 Reagent Cartridge (Illumina, Cat. #20024908).

### Metagenome assembly, binning, and quality checks

Raw metagenomic reads were first trimmed and filtered for contamination using BBTools v38.87 (Bushnell, 2014). Quality filtering excluded adapter contaminants, read regions with scores < 20, and all reads under 50 bp. The resulting paired metagenomic reads were assembled using MetaSPAdes v3.14.0 (Nurk et al., 2017) with kmers specified as 21, 33, 55, and 77. MetaBAT2 v2.15 (Kang et al., 2019) was used to create genome bins from the assembled contigs by first using Seqtk v1.3 (Li, 2018) to remove contigs under 1 kb, then mapping the filtered contigs using BBTools v38.87 (Bushnell, 2014) with default parameters. Samtools v1.10 (Li et al., 2009) was used to sort and index the mapped contigs using default parameters, and BBTools v38.87 (Bushnell, 2014) then generated contig depths for all contigs over 1.5 kb. This contig depth file containing mean and variance of the base coverage depth was used in generating genome bins with MetaBAT2 v2.15 (Kang et al., 2019) with otherwise default parameters. The lineage workflow parameter of CheckM v1.1.3 (Parks et al., 2015) was used to assess completeness and contamination of the binning results to ensure bin quality for downstream analysis.

GTDB-Tk v1.4.0 (Chaumeil et al., 2020) was used to assign taxonomic affiliation to all genome binning results. All bins identified by GTDB-Tk as *Caldarchaeales* were selected for downstream analysis. QUAST v5.0.2 (Gurevich et al., 2013) was used to determine the number of scaffolds, genome size, N50 value, and genomic G + C content. Estimated genome size was determined from CheckM (Parks et al., 2015) completeness and the genome size result from the QUAST analysis. FastANI (Jain et al., 2018) was used to compute ANI values between the five *Caldarchaeales* MAGs and 79 *Caldarchaeales* genomes downloaded from the GTDB release 207_v2.

### Genome annotation and metabolic reconstruction

Prodigal (Hyatt et al., 2010), with the “-p meta” option, was used to predict protein-coding genes in the five *Caldarchaeales* MAGs. All predicted proteins were analyzed with InterProScan (Jones et al., 2014) with default parameters to annotate protein domains and assigned to archaeal clusters of orthologous genes (arCOGs; Makarova et al., 2015b) by eggNOG-mapper v2.1.9 (Cantalapiedra et al., 2021) with default settings. Protein-coding genes were also annotated using the Kyoto Encyclopedia of Genes and Genomes (KEGG) database (Kanehisa and Goto, 2000) tool GhostKOALA v2.2 (Kanehisa et al., 2016) and METABOLIC (Zhou et al., 2022); and annotations for genes of interest were confirmed by the BLASTp (Altschul et al., 1997, 2005) searches that were performed against the following databases: NCBI-nr (Sayers et al., 2021), UniRef100 (Suzek et al., 2015), and UniProtKB reference proteomes plus Swiss-Prot (UniProt Consortium, 2021; E-value cutoff ≤ 1e^−5^). Subcellular localization of peptidases found by METABOLIC against the MEROPS database was predicted by the PSORTb web tool v3.0.3 (Yu et al., 2010). The number of rRNA-coding sequences and presence of 16S rRNA gene in the MAGs were determined with Anvi’o (Eren et al., 2021). The Hidden Markov Model (HMM) profiling function, which utilizes Barrnap,^1^ predicted the locations of rRNA genes. The tRNAscan-SE software v1.3.1 (Chan and Lowe, 2019) was used to determine the number of tRNAs in each MAG. Metabolic pathways were reconstructed based on the hits for key metabolic marker genes and the reference pathways depicted in KEGG (Kanehisa and Goto, 2000) and MetaCyc (Caspi et al., 2020) databases.

### Analysis of CRISPR-Cas systems

CRISPR arrays of repeat-spacer units, and *cas* genes were identified using the combination of web tools CRISPRCasFinder (Couvin et al., 2018), CRISPRone (Zhang and Ye, 2017), CRISPRminer2 (Zhang et al., 2018), and CRISPRCasTyper (Russel et al., 2020). CRISPRone, CRISPRminer2, and CRISPRCasTyper were run with default settings. While running CRISPRCasFinder, the “General” clustering model and the “Unordered” function were used to identify *cas* genes. The CRISPRCasFinder tool has an evidence level rating that ranges from 1 to 4 for detection of CRISPR arrays. Only CRISPR arrays having evidence levels 3 and 4 were included in our analysis. While defining the *cas* locus, CRISPR locus, and CRISPR-Cas locus, and determining the type of a *cas* locus here, we followed published criteria (Zhang and Ye, 2017): (1) a *cas* locus should contain at least three *cas* genes, and at least one of those should belong to the universal *cas* genes for CRISPR adaptation (*cas1* and *cas2*) or the main components of interference module including *cas7*, *cas5*, *cas8*, *cas10*, *csf1*, *cas9*, *cpf1* (Makarova et al., 2015a); (2) CRISPR arrays that are close to each other (≤200 bps) and share very similar repeat sequences were considered to be in the same locus; and (3) the CRISPR(s), together with nearby (within 10,000 bps) *cas* genes, are defined as a CRISPR-Cas locus. The type of each *cas* locus was determined based on type signature *cas* genes listed in Makarova et al., 2015a,b The type-assignment of a *cas* locus was considered confident if it had at least three type-consistent signature *cas* genes, except for type V, which has only one signature gene, *cpf1* (Makarova et al., 2015a; Zhang and Ye, 2017). If a *cas* locus included only one or two type signature *cas* genes, we considered the type-assignment as putative.

### Prediction of putative prophage regions in the MAGs

The VIBRANT v1.2.0 (Kieft et al., 2020) with default settings through the CyVerse Discovery Environment at https://de.cyverse.org/de (Merchant et al., 2016) and VirSorter v1.0.5 (Roux et al., 2015) with default settings against the Viromes database through the DOE Systems Biology KBase at http://kbase.us (Arkin et al., 2018), were used on all contigs of at least 1 kb to identify putative viral genes in the MAGs. All putative viral gene sequences detected by VIBRANT and VirSorter were queried against UniRef100 (Suzek et al., 2015), and UniProtKB reference proteomes plus Swiss-Prot (UniProt Consortium, 2021) databases using the BLAST tool (Altschul et al., 1990) with BLASTp (Altschul et al., 1997, 2005) at https://beta.uniprot.org/blast for functional annotation. Furthermore, these putative viral gene sequences were also functionally annotated by querying them against the NCBI non-redundant protein sequences (nr; Sayers et al., 2021) and IMG/VR Viral Resources v3 (Roux et al., 2021) databases using BLASTp. The BLAST hits with e-values equal to or lower than 1e^−5^ were accepted as statistically significant hits. HHpred, HHblits, and ProtBLAST/PSI-BLAST tools of the MPI Bioinformatics Toolkit (Zimmermann et al., 2018; Gabler et al., 2020) and NCBI CD (Conserved Domains)-Search tool (Lu et al., 2020) were also utilized for further annotation of putative viral genes in our MAGs. The search for auxiliary metabolic genes (AMGs) in the putative prophage regions of the MAGs was carried out based on the list of AMGs provided by Kieft et al. (2020).

VIBRANT uses HMM profiles from three databases: KEGG KoFam, March 2019 release (Kanehisa and Goto, 2000; Aramaki et al., 2020), Pfam (v32; Mistry et al., 2021), and Virus Orthologous Groups (VOG) release 94^2^ to identify and annotate viral proteins (Kieft et al., 2020). On the other hand, VirSorter compares predicted protein sequences to Pfam (v27; Mistry et al., 2021) and to the user-selected viral reference database (Viromes database was chosen for this study) to detect viral-like genes (Roux et al., 2015).

If the protein sequence of a gene found in a putative viral region did not have any hits in KEGG, KoFam, Pfam, VOG, and Viromes databases, and this protein sequence only yielded hits to hypothetical or uncharacterized proteins when BLASTed against UniRef100, UniProtKB reference proteomes plus Swiss-Prot, NCBI nr, and IMG/VR databases, it was then annotated as a hypothetical/uncharacterized protein. We also manually inspected the KEGG, eggNOG-mapper, and InterPro annotations of the MAGs to search for viral hallmark proteins, specifically to identify any viral region not detected by VIBRANT and VirSorter tools. Viral hallmark proteins are necessary for productive infection, such as structural (e.g., capsid, tail, baseplate, portal, coat, spike), terminase, or viral holin/lysin proteins, and their presence is one of the metrics used to capture viral signatures in metagenomes (Roux et al., 2015; Kieft et al., 2020).

### Anvi’o analysis

Pangenomic analyses were performed using Anvi’o v7.1 for three of the MAGs with sufficient genome completeness (>55%): 146_bin.21, 146_bin.25, and 1054_113_bin.10 and their closest relatives in the GTDB release 207_v2 (Table 1). Since two of the MAGs generated from the Chile samples had genome completeness levels lower than 55%, they were excluded from the pangenomic analyses. All of the closest relatives of 146_bin.21, 146_bin.25, and 1054_113_bin.10 in the GTDB displaying completeness levels higher than 55% were included in the analyses. The first pangenomic analysis included 1054_113_bin.10 and three reference genomes that showed >95% ANI (Average Nucleotide Identity) with 1054_113_bin.10 and belonged to the GTDB species *Caldarchaeum subterraneum_B* (GCA_011373365.1, GCA_011364015.1, GCA_011364605.1).

**Table 1:** Genomic features of the five *Caldarchaeales* MAGs.

Bin ID	1054_108_bin.3	1054_113_bin.10	1054_113_bin.2	146_bin.21	146_bin.25
Sample name/source	ET Poppy.17 (Chile)	ET 181107.CE.5 (Chile)	ET 181107.CE.5 (Chile)	100086B (Hawai‘i)	100086B (Hawai‘i)
No. of contigs	167	4	2	21	3
Genome size (Mbp)	0.823684	0.842732	0.632821	1.786704	1.567119
Estimated genome size (Mbp)	1.86	1.19	2.33	1.87	1.6
Largest contig size (Kbp)	12.9	628.7	575.2	371.3	857
Genomic G + C (%)	52.02	51.57	51.96	50.42	62.31
N50 value (bp)	5,339	628,726	575,203	185,085	857,045
No. of protein coding genes	956	910	702	2086	1,624
No. of rRNAs	0	2	0	1	1
Presence of 16S rRNA gene	No	Yes	No	Yes	Yes
No. of tRNAs	19	27	7	35	44
No. of genes annotated by KO	498 (52.1%)	539 (59.2%)	353 (50.3%)	1,054 (50.5%)	868 (53.4%)
Completeness (%)	44.34	70.87	27.18	95.63	98.06
Contamination (%)	0	0	0	0.97	0
Accession number	JANVYS000000000	JANVYU000000000	JANVYT000000000	JANVYQ000000000	JANVYR000000000
SeqCode name	N/A	N/A	N/A	*Pelearchaeum maunauluense*	*Calditenuis fumarioli*

The second pangenomic analysis included 146_bin.25 and two reference genomes that showed >95% ANI with 146_bin.25 and belonged to the GTDB species JGI-OTU-1 sp011364265 (GCA_011364265.1, GCA_011369755.1). Lastly, the third pangenomic analysis included 146_bin.21 and two reference genomes that showed approximately 80% ANI with 146_bin.21 and belonged to the GTDB species WAQC01 sp015520425 (GCA_015520425.1, GCA_015522085.1). Due to relative incompleteness of closely related MAGs in GTDB, we were only able to use very few genomes in the pangenomic comparisons. To ensure continuity, previous protein-coding gene annotations completed by Prodigal were first processed using “anvi-script-process-genbank” and imported into the Anvi’o contigs databases using the “—external-gene-calls” flag and “anvi-import-functions” program. After the pan-genomes were generated using the “anvi-pan-genome” program, the ANIb values between query and reference genomes were imported using PyANI from the “anvi-compute-genome-similarity” program within the Anvi’o suite.

### Phylogenomic analyses

The Genome Taxonomy Database Toolkit (GTDB-Tk) version 2.1.1 was used alongside database release 207_v2 to classify the five MAGs presented here. The resulting multiple sequence alignment (MSA) containing 53 concatenated and conserved single-copy archaeal marker genes (10,153 characters) in 3492 archaeal genomes, which included the five MAGs generated in this study, was used as an input to perform a model test in the IQ-Tree program (version 2.1.4-beta; Minh et al., 2020). A maximum likelihood phylogenomic tree was constructed using the best model chosen based on the AIC and BIC criteria (LG + F + G4), with 1,000 ultra-rapid bootstrap replicates. The resulting tree was uploaded to iTOL (Interactive Tree of Life; Letunic and Bork, 2021) to collapse known clades, and edited in Inkscape for esthetics only.

### Calculation of the abundance and distribution of *Caldarchaeales* from published datasets

Barrnap v0.9^3^ was used to identify and extract 16S rRNA gene sequences from three of our five *Caldarchaeales* genomes (1054_113_bin.10, 146_bin.21, 146_bin.25). To detect the presence of *Caldarchaeales* across diverse habitats, all three 16S rRNA gene sequences were used as queries for the Integrated Microbial NGS Platform (IMNGS; Lagkouvardos et al., 2016) to perform a Paraller similarity search (97% similarity), which analyzes all available prokaryotic 16S rRNA amplicon experimental datasets from the International Nucleotide Sequence Database Collaboration (INSDC). The INSDC includes datasets submitted from the DNA Data Bank of Japan (DDBJ), European Molecular Biology Laboratory’s European Bioinformatics Institute (EMBL-EBI), and National Center for Biotechnology Information (NCBI). The IMNGS result provided the relative abundance of reads and environmental descriptors for each hit, but data for location coordinates were searched manually in the NCBI SRA database or original literature due to inconsistencies in location reporting. The relative abundance was calculated by dividing the number of sequences identified by IMNGS to be >97% identical to the query 16S rRNA gene sequences by the total number of sequences in each 16S rRNA amplicon survey. All non-environmental 16S rRNA amplicon surveys (e.g., oral, skin, gut) were removed from downstream analysis, and amplicon sequences with identical location coordinate data were combined. The 16S rRNA gene sequences of our three *Caldarchaeales* MAGs were also searched against the Silva 16S rRNA database to obtain GenBank accession numbers for all hits. These GenBank accession numbers were downloaded and parsed using custom Python scripts to extract environmental information and coordinate location data. GenBank accessions containing location coordinate data were used in the geographic analysis, and again, identical locations were combined. Environmental features and location coordinates data for each hit were visualized in R using the ggplot2 (Wickham, 2016) and maps (Becker et al., 2022) packages. Relative abundance data were visualized for all 16S rRNA amplicon surveys found using IMNGS.

## Results and discussion

### Diversity, abundance, and distribution of the *Caldarchaeales*

**Figure 1:**
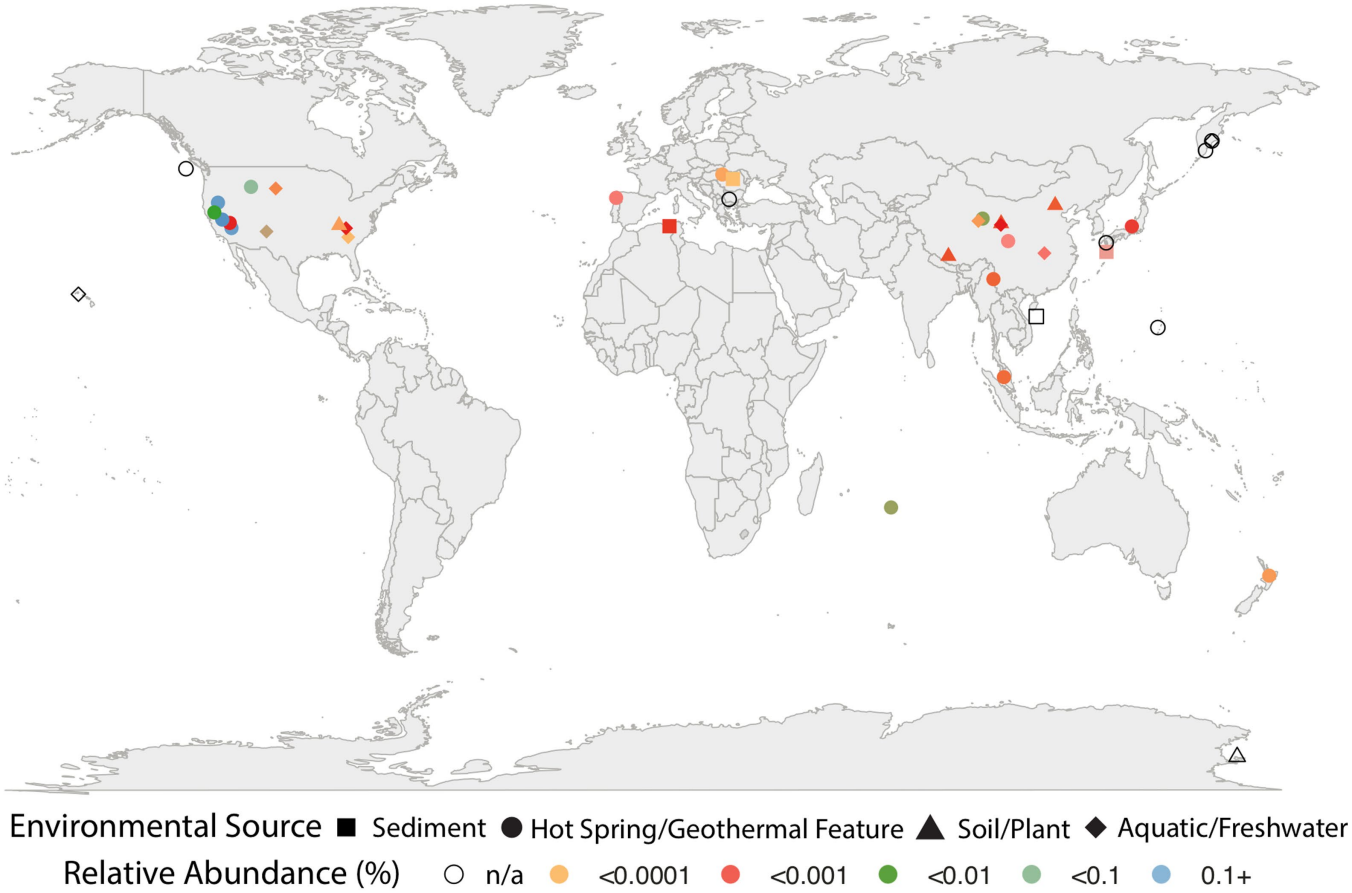
Environmental distribution of *Caldarchaeales* based on presence of 16S rRNA sequences identified in 500,048 samples from publicly available database. The symbols in various colors represent sequences from IMNGS and correspond to relative abundance (see key); symbols outlined in black represent sequences from the Silva 16S database, and do not include abundance information.

Searches against the IMNGS and Silva 16S rRNA databases revealed that the *Caldarchaeales* are represented in a diverse array of features, including hot springs, marine geothermal features, lake and ocean sediments, soil, and other aquatic systems. They were also determined to be present in low relative abundance (<0.0001%) in plants such as *Populus deltoides*, fish gut metagenomic studies, and in surveys of human skin, oral, and gut microbial communities. Although they appear to inhabit a wide array of ecological niches, *Caldarchaeales* are especially prevalent in hot springs and other geothermal features (Figure 1; Supplementary Table S1). We cannot exclude the possibility that some of the unusual habitats the *Caldarchaeales* are found might be attributed to barcode misassignments or contaminations in the samples we obtained from these public databases. The Great Basin and Yellowstone regions of the United States host the greatest relative abundance of these archaea. These regions likely provide optimal conditions for thermophilic archaea since the abundance of affiliated sequences in all other features and areas is at least 100-fold less than in US hot springs. It should also be noted that in some soil and sediment environments, temperatures can be similar to those in many hot springs, such as a fumarolic soil sample from Antarctica in which the temperature was 65°C (Herbold et al., 2014). However, there is a possibility that primer bias could have also contributed to higher abundances of these archaea in some of the samples. Additional sampling and metagenomic studies will provide more insights into the geographical distribution of the *Caldarchaeales*.

### General genomic features and phylogenomic placement

General features of the five MAGs along with genome completeness and contamination estimates are shown in Table 1. GTDB-Tk classification of the MAGs resulted in one from Hawai‘i (146_bin.21) being assigned species-level novelty in the GTDB-designated genus WAQC01, in the family *Caldarchaeaceae*, order *Caldarchaeales*. In the GTDB release 207_v2, only two genomes represent WAQC01, and these are MAGs generated from hydrothermal deposits in the subsurface of a submarine volcano in the Pacific Ocean (Reysenbach et al., 2020). The other MAG from Hawai‘i (146_bin.25) was assigned to the GTDB species JGI-OTU-1 sp011364265 in the GTDB genus JGI-OTU-1, family HR02, order *Caldarchaeales*. It forms a sister-level relationship with *Candidatus* “Calditenuis aerorheumensis.” Three MAGs from Chile were assigned to the GTDB species *Caldarchaeum subterraneum B* in the family *Caldarchaeaceae*, order *Caldarchaeales*.

**Figure 2:**
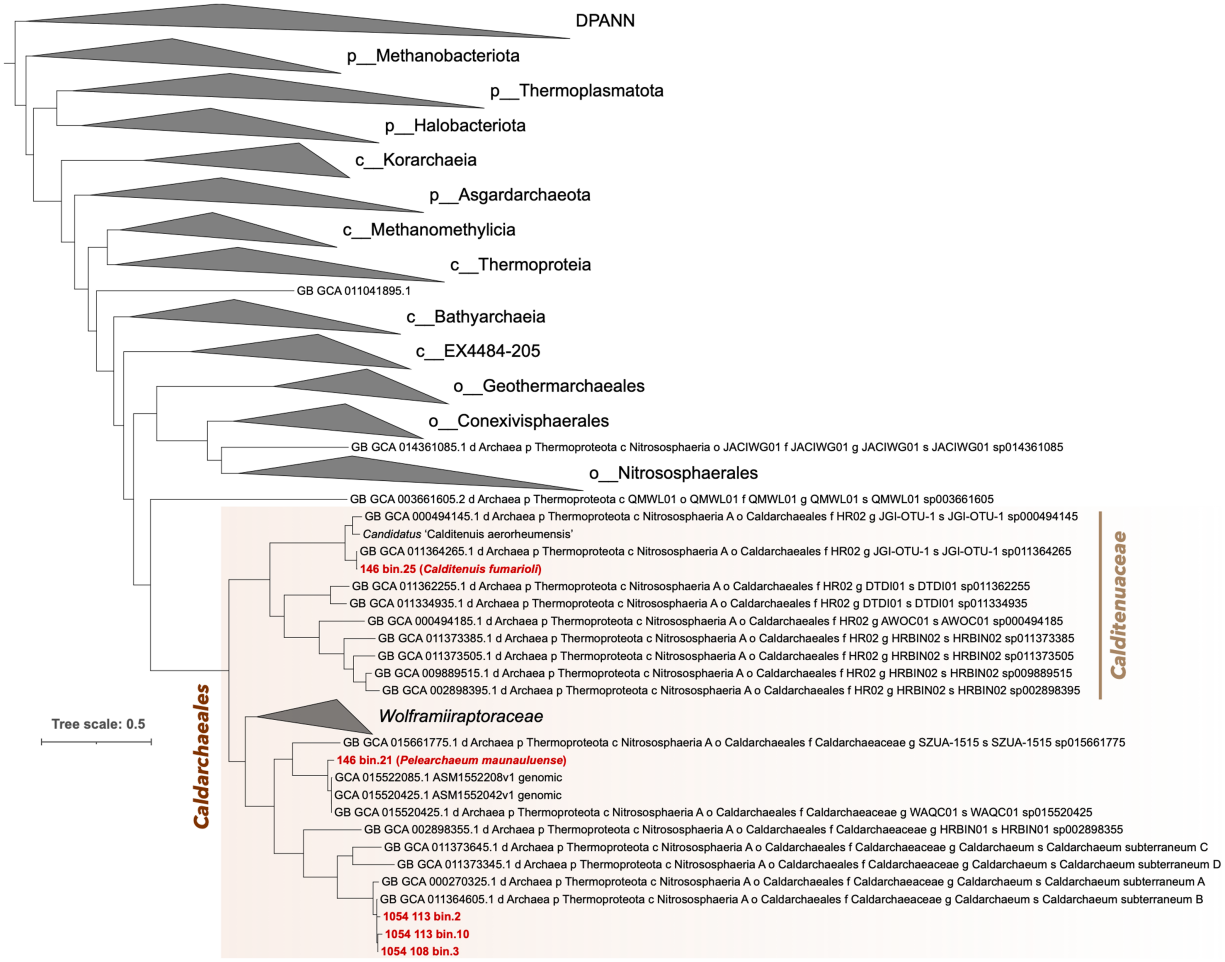
Phylogenomic placement of five *Caldarchaeales* MAGs alongside representative reference archaeal genomes obtained from the GTDB release 207_v2. Prefixes in taxa names indicate the following: p, phylum, c, class, o, order, f, family, g, genus, and s, species. The tree was rooted with DPANN archaea. Known groups of archaea were collapsed and shown as triangles. The order *Caldarchaeales* is highlighted with a light blue background. Full tree containing all taxa is available as Supplementary Data File 1.

IQ-Tree phylogenomic inference of the five MAGs alongside the archaeal backbone tree from the GTDB release 207_v2, shows the three bins from Chile in a clade with *Ca. C. subterraneum* (Figure 2). 146.bin.25 groups closely with members of family HR02 and forms a sister-level relationship with a SAG from Great Boiling Spring (Rinke et al., 2013). It is interesting to note that 146_bin.21 forms a monophyletic clade with two deep-sea hydrothermal vent-associated lineages (Reysenbach et al., 2020) despite it coming from a terrestrial environment. More importantly, we obtained a monophyletic clade comprising all *Caldarchaeales* as sister to the clade comprising the orders *Geothermarchaeales* (previously *Candidatus* Geothermarchaeota)*, Conexivisphaerales* and *Nitrososphaerales* (formerly classified as the phylum Thaumarchaeota).

### Description of new family, genera, and species

Based on the SeqCode guidelines to assign valid names to microbial genomes with relatively high completeness and low contamination, both MAGs from Hawai‘i can be given valid genus and species names as both are above 90% completeness, below 5% contamination, and have small numbers of contigs (Hedlund et al., 2022; Whitman et al., 2022). Here, we propose the names *Pelearchaeum maunauluense*^Ts^ gen. Nov., sp. nov. for 146_bin.21 after Pele, the goddess of volcanoes and fire, and *Calditenuis fumarioli* ^Ts^ sp. nov. for 146_bin.25, which belongs to the same genus as *Candidatus* “Calditenuis aerorheumensis” (ANI value of 81.07% between them; Figure 2). Due to monophyletic grouping of 146_bin.25 with 10 other members (Figure 2), we also propose to name the clade (GTDB family designation HR02) as *Calditenuaceae* and designate *Calditenuis fumarioli*^Ts^ as type species. A list of proposed names, etymologies, and nomenclatural types are shown in Table 2. Hereafter, we will refer to 146_bin.21 as *P. maunauluense* and 146_bin.25 as *C. fumarioli*.

**Table 2:** A table of proposed names.

Proposed taxon	Etymology	Nomenclatural type
Family *Calditenuaceae*	[Cal.di.te’nu.a.ce’ae] N.L. fem. pl. n. *Calditenuis* is the type genus of the family; −aceae ending to denote a family; N.L. fem. Pl. n. *Calditenuaceae*, the Calditenuis family	Genus *Calditenuis*
Genus *Calditenuis*	[Cal.di.te’nu.is] L. masc. Adj. *caldus*, warm; L. masc./fem. Adj. *tenuis*, thin, slender; N.L. masc. n. *Calditenuis*, a warm and slender organism.	Species *Calditenuis fumarioli*^Ts^
Genus *Pelearchaeum*	[Pe.le.ar.chae’um] N.L. fem. n. *Pele*, name of the goddess of volcanoes and fire in Hawaiian mythology, N.L. neut. n. *archaeum*, an archaeon.	Species *Pelearchaeum maunauluense*^Ts^
Species *Calditenuis fumarioli*^Ts^	[fu.ma.ri.o’li] N.L. gen. n. *fumarioli*, of a fumarole, referring to the source of the nomenclatural type.	Genomic assembly: GCA_030059665.1^Ts^
Species *Pelearchaeum maunauluense*^Ts^	[mau.na.u.lu.en’se] N.L. neut. Adj. maunauluense, of Mauna Ulu volcano in Hawaii. In Hawaiian, ulu means to grow or sprout.	Genomic assembly: GCA_030059675.1^Ts^

### Metabolic features

**Figure 3:**
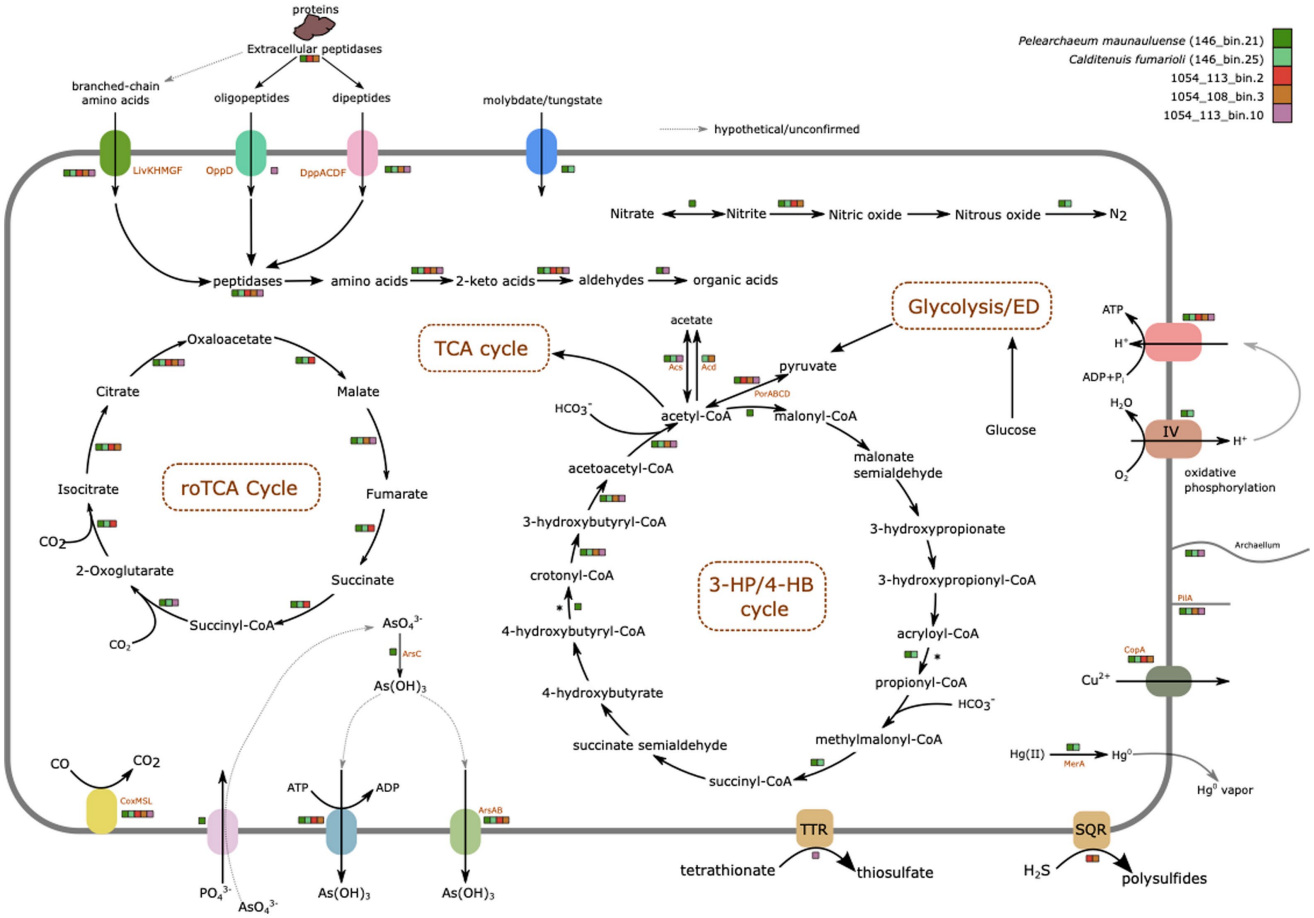
Overview of major metabolic features identified in the five MAGs from Hawai’i and Chile. MAGs are color-coded to indicate whether presence of genes encoding enzymes involved in these depictions have been identified in their genomes or not. Dotted lines surrounding the names of the pathways identified indicate partial nature of the pathways in some MAGs due to incomplete nature of the MAGs. The figure was manually drawn using Inkscape.

Metabolic features inferred from the genomes of the five MAGs are summarized in Figure 3; Supplementary Tables S2, S3, S10. GhostKOALA and eggNOG-mapper annotations of all coding sequences can be found in Supplementary Tables S4, S5, respectively. Their metabolic potentials are discussed in greater details below.

#### Central carbon metabolism

Only *P. maunauluense*, *C. fumarioli*, 1054_113_bin.2, and 1054_108_bin.3 include at least one marker gene of the glycolysis (Embden-Meyerhof-Parnas) pathway (Figure 3). *Pelearchaeum maunauluense*, 1054_113_bin.2, and 1054_108_bin.3 possess the metabolic potential for the Branched Entner-Doudoroff pathway to utilize glucose. These MAGs include two marker genes of the Branched ED pathway: genes encoding 2-dehydro-3-deoxygluconokinase/2-dehydro-3-deoxygalactonokinase and glycerate 2-kinase (Siebers et al., 2004; Kehrer et al., 2007; Bräsen et al., 2014; Sutter et al., 2016). The 1054_108_bin.3 and 1054_113_bin.10 lack both oxidative and non-oxidative phases of the pentose phosphate pathway (PPP), whereas *P. maunauluense*, *C. fumarioli*, and 1054_113_bin.2 possess incomplete oxidative PPP and lack the non-oxidative phase of PPP. It was reported that the Ribulose Monophosphate Pathway (RuMP) could substitute for the missing non-oxidative PPP in some archaea (Orita et al., 2006). *Pelearchaeum maunauluense* encodes genes for the bifunctional enzyme—3-hexulose-6-phosphate synthase (*hps*) and 6-phospho-3-hexuloisomerase (*phi*)—suggesting it can conduct Ru5P synthesis by RuMP, despite lacking the non-oxidative PPP.

The genes encoding fructose-1,6-bisphosphatase and phosphoenolpyruvate carboxykinase enzymes are the marker genes for the gluconeogenesis pathway (Dombrowski et al., 2018). A gene encoding fructose-1,6-bisphosphate aldolase/phosphatase (type V fructose-1,6-bisphosphatase) was identified in *P. maunauluense*, *C. fumarioli*, and 1054_113_bin.10. However, another key enzyme involved in gluconeogenesis, phosphoenolpyruvate carboxykinase was found only in the *P. maunauluense* genome. Yet, it is known that the function of this enzyme in gluconeogenesis pathway could be fulfilled by phosphoenolpyruvate synthase (pyruvate, water dikinase) or pyruvate:phosphate dikinase (Bräsen et al., 2014). Only *P. maunauluense*, *C. fumarioli*, and 1054_113_bin.10 encode pyruvate:phosphate dikinase enzyme, which could be utilized for gluconeogenesis in the absence of phosphoenolpyruvate carboxykinase. In addition to the type V fructose-1,6-bisphosphatase, we found type IV fructose-1,6-bisphosphatase (Stec et al., 2000; Verhees et al., 2002; Bräsen et al., 2014), which shows both fructose-1,6-bisphosphatase and inositol monophosphatase activities, in *P. maunauluense* and *C. fumarioli*.

All bins display the potential to decarboxylate pyruvate to acetyl-coenzyme A and link glycolysis with the oxidative tricarboxylic acid (oTCA) cycle (Figure 3). All except 1054_113_bin.10 encode both pyruvate ferredoxin/flavodoxin oxidoreductase (POR) and pyruvate dehydrogenase (PDH) complex. 1054_113_bin.10 only encodes PDH complex. POR catalyzes the decarboxylation of pyruvate during fermentation in anaerobes, while PDH complex catalyzes the same reaction during respiration in aerobes (Müller et al., 2012; Gould et al., 2019). Anaerobic and aerobic conditions are known to generally fluctuate in oxygen-limited environments such as deep subsurface aquifers and surface sediments. Hence, the presence of both POR and PDH complex could help microorganisms adapt to oxygen fluctuations as suggested earlier (Fang et al., 2021). While *P. maunauluense* and *C. fumarioli* encode genes for complete oTCA cycle, 1054_108_bin.3, 1054_113_bin.10, and 1054_113_bin.2 lack some of the genes. However, all five MAGs encode citrate synthase, which is one of the key enzymes of the oTCA cycle (Danson, 1993), suggesting that the cycle may be present in all the bins.

It is known that β-oxidation of fatty acids involves at least four enzymes: acyl-CoA dehydrogenase, crotonase, 3-hydroxyacyl-CoA dehydrogenase and acetyl-CoA acetyltransferase (Wang et al., 2019, 2021). Acyl-CoA dehydrogenase, fused enoyl-CoA hydratase and 3-hydroxybutyryl-CoA dehydrogenase enzyme, and acetyl-CoA C-acetyltransferase were found in *P. maunauluense*, *C. fumarioli*, 1054_108_bin.3, and 1054_113_bin.10. However, 1054_113_bin.2 encodes only acyl-CoA dehydrogenase. It was suggested that the enzymes involved in fatty acid β-oxidation could also be used for synthesizing fatty acids in *Archaea* (Dibrova et al., 2014). Hence, *P. maunauluense*, *C. fumarioli*, 1054_108_bin.3, and 1054_113_bin.10 exhibit metabolic capacity for utilization of lipids as carbon and energy sources and fatty acid biosynthesis. In addition, the presence of numerous binding/transport proteins for branched chain amino acids/dipeptides/oligopeptides and several aminotransferases and intracellular peptidases in all the MAGs indicates the capability to acquire amino acids and peptides from the surrounding environment and utilize them as a carbon and energy source. Some MAGs also encode a few genes for putative extracellular peptidases, implying the potential to secrete peptidases, degrade detrital proteins outside of the cell and play a role in protein remineralization (Figure 3).

Presence of xylulose kinase genes in 1054_108_bin.3 and 1054_113_bin.10 suggests the capability to degrade xylose, which is the main constituent of xylan polymers, which are the second most abundant group of polysaccharides in nature and the major component of plant cell walls (Beg et al., 2001; Knapik et al., 2019). Recently, a *Caldarchaeales* archaeon grown in enrichment culture in the lab was shown to encode xylose isomerase and xylulose kinase, use xylose as its preferred carbohydrate monomer, and was proposed to contribute to the fermentative degradation of lignocellulose at Great Boiling Spring, Nevada (Buessecker et al., 2022). The presence of similar genes in the two MAGs here from El Tatio hot springs suggests they may also be able to degrade xylose or similar molecules.

#### Fermentation

The detection of *acd* gene encoding ADP-forming acetyl-CoA synthetase (Acd) in *C. fumarioli* and 1054_108_bin.3 indicates the capacity for acetate fermentation even though these MAGs lack the classical phosphate acetyltransferase (Pta)—acetate kinase (Ack) pathway for acetate production (He et al., 2016). Acd is a novel enzyme that catalyzes the conversion of acetyl-CoA to acetate and couples this reaction with the synthesis of ATP via the mechanism of substrate level phosphorylation in *Archaea* and eukaryotic protists (Schönheit and Schäfer, 1995; Musfeldt and Schönheit, 2002). On the other hand, all acetate-forming *Bacteria* use classical Pta-Ack pathway for acetate formation and ATP synthesis except a few members, such as *Chloroflexus aurantiacus* (Schmidt and Schönheit, 2013) and *Propionibacterium acidipropionici* (Parizzi et al., 2012), which were suggested to use Acd for acetate formation based on the proteomic and biochemical evidence.

*P. maunauluense*, *C. fumarioli*, and 1054_113_bin.10 were also found to encode for AMP-forming acetyl-CoA synthetase (Acs) involved in acetate utilization by converting acetate to acetyl-CoA (Ingram-Smith et al., 2006), which suggests the possibility of both acetate production and consumption for *C. fumarioli*. However, Acs might function in the reverse direction, as well. In a recent study, acetogenic activity of both Acd and Acs is demonstrated with the enzyme assays providing strong evidence to conclude that Acs could also catalyze the conversion from acetyl-CoA to acetate in anaerobic methanotrophic archaea (Yang et al., 2020). Taken together, these findings imply that some members of the *Caldarchaeales* could have a metabolic capacity to grow as acetogens and play a role in organic carbon cycling in their ecosystems. None of the MAGs contain the gene encoding aldehyde dehydrogenase responsible for the conversion of acetate to acetaldehyde in the ethanol fermentation pathway, and the gene encoding for alcohol dehydrogenase, which generates ethanol from acetaldehyde, is only found in 146_bin25. Hence, it seems likely that none of the MAGs has the metabolic capacity to perform ethanol fermentation.

#### Carbon fixation

The absence of ATP citrate (pro-S)-lyase and citryl-CoA synthetase/citryl-CoA lyase was previously reported in *Caldarchaeales* genomes, indicating the lack of the reductive TCA Cycle (rTCA; Hua et al., 2018). In our study, genes encoding ATP citrate (pro-S)-lyase and citryl-CoA synthetase were not recovered from any of the MAGs. Citryl-CoA lyase was only observed in the *C. fumarioli* genome. The lack of key enzymes indicates the absence from our MAGs of the rTCA cycle.

An alternative pathway for carbon fixation, referred to as reversed oxidative TCA cycle (roTCA), may exist in *Caldarchaeales* (Hua et al., 2018). This pathway is a version of the rTCA cycle. In the roTCA cycle, citrate synthase replaces ATP-citrate lyase (or citryl-CoA synthetase / citryl-CoA lyase) of the rTCA cycle (Mall et al., 2018; Nunoura et al., 2018). The roTCA cycle was shown to operate in two obligately anaerobic bacteria, *Desulfurella acetivorans* (Mall et al., 2018) and *Thermosulfidibacter takaii* (Nunoura et al., 2018). It was also revealed that the direction of roTCA cycle was controlled by the availability of organic vs. inorganic carbon source(s), reflecting the adaptation of microorganisms to fluctuating concentrations of carbon sources (Mall et al., 2018; Nunoura et al., 2018). Furthermore, in the thermophilic sulfur-reducing Deltaproteobacterium *Hippea maritima*, the roTCA cycle was driven by high partial pressures of carbon dioxide (Steffens et al., 2021). Given that citrate synthase was identified in all MAGs analyzed in our study, and ATP-citrate lyase (or citryl-CoA synthetase/citryl-CoA lyase) is absent from all MAGs, we propose that *Caldarchaeales* lineages represented by these MAGs likely harbor a metabolic potential to carry out the roTCA cycle for CO_2_ fixation as an alternative to the canonical rTCA cycle.

Previously, the dicarboxylate/4-hydroxybutyrate (DC/4HB; Nunoura et al., 2011) and the 3-hydroxypropionate/4-hydroxybutyrate (3HP/4HB; Beam et al., 2016) cycles were proposed to be present in *Caldarchaeales*. *Pelearchaeum maunauluense* genome contains two genes encoding key enzymes for the 3-hydroxypropionate/4-hydroxybutyrate (3HP/4HB) cycle: 4-hydroxybutyryl-CoA dehydratase and acryloyl-coenzyme A reductase (Berg et al., 2010; Hügler and Sievert, 2011; Könneke et al., 2014; Ruiz-Fernández et al., 2020), whereas *C. fumarioli* genome harbors only one key enzyme, acryloyl-coenzyme A reductase. 4-hydroxybutyryl-CoA dehydratase is also the key enzyme of the DC/4HB cycle. Yet, the gene encoding for the other key enzyme of the DC/4HB cycle, 4-hydroxybutyrate—CoA ligase was not found in any of the MAGs. Also, we did not recover any marker genes for the 3HP/4HB cycle in the MAGs from Chile. Despite the occurrence of some marker genes for the 3HP/4HB and DC/4HB cycles, we cannot assert that these cycles are functional considering that they are incomplete in *P. maunauluense* and *C. fumarioli* genomes.

#### C1 and oxygen metabolism

Multiple copies of aerobic carbon monoxide dehydrogenase large subunit (CoxL), aerobic carbon monoxide dehydrogenase medium subunit (CoxM), and aerobic carbon monoxide dehydrogenase small subunit (CoxS) were found in all MAGs. Also, all MAGs likely derive from organisms capable of aerobic respiration since they encode cytochrome c oxidase subunits: *P. maunauluense* has *coxA*, *coxB*, *coxC*, and *coxAC*; *C. fumarioli* has *coxA*, *coxB*, *coxD*, and *coxAC*; 1054_113_bin.2 has *coxC*; 1054_108_bin.3 has also only *coxC*; and 1054_113_bin.10 has *coxA* and *coxB*. Taken together, these findings imply the ability to grow chemolithotrophically via carbon monoxide (CO) oxidation coupled to oxygen reduction by cytochrome c oxidase complex. CO is a common gas in hot volcanic environments and hot springs since it is present in volcanic emissions and may be produced during thermal and photochemical decomposition of organic matter, or as a byproduct by some thermophilic anaerobes (Sokolova et al., 2009; Kochetkova et al., 2011; Baker et al., 2016). It was suggested that the ability to utilize CO is widespread among aerobic bacteria, and certain archaea (e.g., *Pyrobaculum* and *Sulfolobus*) likely carry out aerobic CO oxidation as well (King, 2003a). It was also proposed that CO could serve as an energy supplement when organic substrates are deficient, which is consistent with the observation that CO utilization is associated with microbial community development on Hawaiian volcanic deposits (King, 2003b). Previously, potential aerobic type carbon monoxide dehydrogenases were detected in *Caldarchaeales* (Nunoura et al., 2011; Beam et al., 2016; Hua et al., 2018), and it was speculated that *Caldarchaeales* could oxidize CO aerobically as an energy supplement in the case of limited organic substrates and the supplied energy might contribute to biomass by coupling with carbon fixation through the roTCA cycle (Hua et al., 2018).

#### Sulfur cycling

1054_108_bin.3 and 1054_113_bin.2 encode sulfide:quinone oxidoreductase, indicating the capacity to use hydrogen sulfide as an electron donor for chemolithotrophy. The presence of dissimilatory sulfite reductase (*dsrAB*) genes was previously reported in several *Caldarchaeales* genomes (Rinke et al., 2013; Hua et al., 2018). However, we did not recover *dsrA* and *dsrB* genes from our MAGs, which suggests they may represent organisms incapable of dissimilatory sulfite reduction. Moreover, multiple copies of the gene encoding the tetrathionate reductase subunit A were detected in 146_bin21, while all subunits of the tetrathionate reductase are present in 1054_113_bin.10, suggesting the organism from which this MAG was derived might conduct tetrathionate respiration, consistent with a previous study reporting a tetrathionate reductase complex in *Caldarchaeales* (Beam et al., 2016). In addition, Sanchez-Garcia et al., 2019 revealed that in the liquid geyser mounds at El Tatio geothermal fields, microbial metabolism is dominated by the autotrophic Calvin cycle, along with lesser sulfur and iron chemolithotrophic pathways, and iron and sulfur oxidizers and sulfate reducers are abundant in the liquid geyser mounds. Sulfate was detected in both water and sinter deposit samples of the liquid geyser mounds with the water collected from the liquid mound containing higher concentrations of sulfate (346 μg g^−1^) compared to its sinter deposits (14 μg g^−1^; Sanchez-Garcia et al., 2019). The findings of this previous study and the presence of genes involved in dissimilatory sulfur metabolism in the MAGs from El Tatio indicate that *Caldarchaeales* members might be also contributing to the sulfur cycling at El Tatio along with bacterial sulfur oxidizers and sulfate-reducing bacteria.

#### Nitrogen cycling

*Pelearchaeum maunauluense* is the only MAG encoding *narG*, *narZ*, *nxrA*; nitrate reductase/nitrite oxidoreductase, alpha subunit and *narY*, *nxrB*; nitrate reductase/nitrite oxidoreductase, beta subunit. This indicates the organism represented by *P. maunauluense* might perform the first step of denitrification, which is reduction of nitrate (NO_3_^−^) to nitrite (NO_2_^−^), and that it may also oxidize nitrite to nitrate (the second step of nitrification). Under anaerobic conditions, nitrite oxidoreductase functions as a nitrate reductase, catalyzing the reverse reaction. *Pelearchaeum maunauluense*, *C. fumarioli*, 1054_113_bin.2, and 1054_108_bin.3 encode the *nirK*; nitrite reductase (NO-forming), which suggests the organisms represented by these MAGs might perform anaerobic respiration by reducing nitrite (NO_2_^−^) to nitric oxide (NO; the second step of denitrification). However, nitric oxide reductase involved in the reduction of NO to nitrous oxide (N_2_O; the third step of denitrification) were not identified in any of the MAGs. Since highly toxic NO is not likely to be reduced enzymatically, it could be abiotically reduced by iron as previously put forward by Kozlowski et al. (2016). This notion is supported by the studies reporting (a) the detection of iron oxides and dominance of microorganisms participating in the iron and sulfur geochemical cycles in liquid geyser mounds at El Tatio geothermal fields (Sanchez-Garcia et al., 2019) and (b) high concentrations of iron in basaltic rocks and their associated fumaroles (Cockell et al., 2019). *Pelearchaeum maunauluense* and *C. fumarioli* genomes encode *nosZ*; nitrous-oxide reductase, which suggests that the organisms represented by these MAGs might conduct anaerobic respiration by reducing N_2_O to nitrogen gas (N_2_; the fourth and last step of denitrification).

### Versatile metabolic potentials of *Caldarchaeales* as adaptive mechanisms for life in extreme environments

The presence of some key marker genes for the 3HP/4HB and DC/4HB cycles in the MAGs from Hawai‘i hints at the capacity to fix carbon. However, these cycles are not complete. All MAGs from Hawai‘i and Chile also possess metabolic potential for roTCA cycle, but only further experimental validation will determine their functionality. Metabolic pathway predictions suggest the potential denitrification and tetrathionate reduction pathways might enable these lineages to respire diverse compounds other than oxygen. All MAGs except 1054_113_bin.10 encode reverse gyrase, a marker gene for hyperthermophiles, which is consistent with the environmental conditions of the habitats from which the MAGs were recovered. The occurrence of genes involved in aerobic and anaerobic respiration in the MAGs indicates a facultative anaerobic lifestyle, which would enable growth in anoxic environments. We also determined that the MAGs contain several genes implicated in oxidative stress tolerance, such as those for superoxide dismutase, thioredoxin reductase, and peroxiredoxin, which could be used to alleviate oxidative damage.

Some of our MAGs also possess genes involved in arsenic detoxification and resistance to mercury (Figure 3). Only *P. maunauluense* genome encodes genes capable of encoding arsenate reductase (*arsC*), though all other MAGs encode the gene for the ArsR family transcriptional regulator (*arsR*). Evidence for genes that encode the arsenical pump membrane protein (*arsB*) and arsenite/tail-anchored protein-transporting ATPase (*arsA*) were detected in all MAGs except 1054_113_bin.10. As for resistance to mercury, *P. maunauluense* and *C. fumarioli* genomes encode the gene for mercuric reductase (*merA*). The presence of arsenic and mercury in fumarolic rocks of Hawai‘i is currently unknown. Hot springs are known to contain various levels of arsenic and at El Tatio, the concentrations can sometimes reach 40–50 mg/L of hot spring water, which is among one of the highest concentrations measured in any natural habitat (Wang et al., 2018) and therefore presence of transporters to pump out various arsenic related compounds such as arsenous acid [As(OH)_3_] appear to indicate that *Caldarchaeales* in these habitats may be actively utilizing these detoxification systems for their survival.

### Tungstoenzymes and tungstate transporters

A recent study has reported the enrichment and stable laboratory growth of *Caldarchaeales* from sediments of Great Boiling Spring (Nevada, United States), designated *Wolframiiraptor gerlachensis*, which belongs to the GTDB family NZ13-MGT (Buessecker et al., 2022). Cultivation experiments and the analysis of a MAG from *W. gerlachensis*, which includes six annotated tungsten (W)-dependent ferredoxin oxidoreductases that could play central roles in anaerobic carbohydrate degradation, have suggested that tungsten could be essential for carbohydrate metabolism, and that it may play a critical role in one or more of the annotated W-dependent ferredoxin oxidoreductases, thereby indicating that the growth of *W. gerlachensis* requires the presence of tungsten, a biologically rare trace metal (Buessecker et al., 2022). In addition, the evolutionary analysis of 78 MAGs representing four genera and 11 species of the GTDB family NZ13-MGT, designated as *Wolframiiraptoraceae*, has demonstrated that putative tungstate transporters and tungstoenzymes were ancestral in this lineage. Furthermore, homologs of 3 W-dependent ferredoxin oxidoreductases: aldehyde:ferredoxin oxidoreductases (AOR), glyceraldehyde-3-phosphate:ferredoxin oxidoreductases (GAPOR), and formaldehyde:ferredoxin oxidoreductases (FOR) are widely distributed in the family *Wolframiiraptoraceae* genomes, and sparse in other *Caldarchaeales*.

Also, the majority of the *Wolframiiraptoraceae* genomes has been detected to encode Tup ABC tungstate transporter system, whereas other ABC tungstate transporter systems: Wtp and Mod have only been identified in two species in the family *Wolframiiraptoraceae* (Buessecker et al., 2022). Given the significant role of tungsten in sugar and peptide catabolism within the *Caldarchaeales* (Buessecker et al., 2022), we searched for tungstate transporters and tungsten-associated enzymes in the five MAGs. Five AOR homologs were identified, one in 1054_113_bin.10, and four genes in *P. maunauluense*, three of which are fragmented. However, we did not recover any GAPOR or FOR homologs in the MAGs. *Pelearchaeum maunauluense* genome also encodes three subunits of the Wtp molybdate/tungstate transporter system, but no tungstate transporter system was annotated in 1054_113_bin.10, possibly due to incomplete genomes (Figure 3). Intriguingly, *C. fumarioli* includes three subunits of the Wtp molybdate/tungstate transporter system and an adjacent gene encoding a molybdate transport system regulatory protein, but no W-dependent ferredoxin oxidoreductases. The AOR homologs encoded in *P. maunauluense* and 1054_113_bin.10 may be involved in the protein catabolism by converting aldehydes, which were produced from 2-Keto acids by ferredoxin oxidoreductases to organic acids and also in the Branched Entner-Doudoroff pathway; GAPOR is only known to function in unusual Embden-Meyerhof pathways in which it converts glyceraldehyde-3-phosphate to 3-phosphoglycerate, replacing glyceraldehyde-3 phosphate dehydrogenase and phosphoglycerate kinase enzymes used in the canonical Embden-Meyerhof pathway (Mukund and Adams, 1995; Bevers et al., 2005). *Pelearchaeum maunauluense*, *C. fumarioli*, 1054_108_bin.3, and 1053_113_bin.10 encode both glyceraldehyde-3 phosphate dehydrogenase and phosphoglycerate kinase enzymes, which might explain the absence of a GAPOR homolog from these genomes.

### Analysis of prophage regions

Previously, integrated mobile genetic elements (IMGEs) that include bacterial insertion sequence (IS)-like transposons, prophages, and cryptic integrated elements were reported in several *Caldarchaeales* genomes (Nunoura et al., 2011; Hua et al., 2018; Krupovic et al., 2019). In line with these studies, we also found putative prophage regions and genes encoding transposases in our MAGs, suggesting the MAGs might harbor IMGEs. Prophage-related genes and transposases identified in the MAGs are shown in Supplementary Tables S6, S7, respectively.

**Figure 4:**
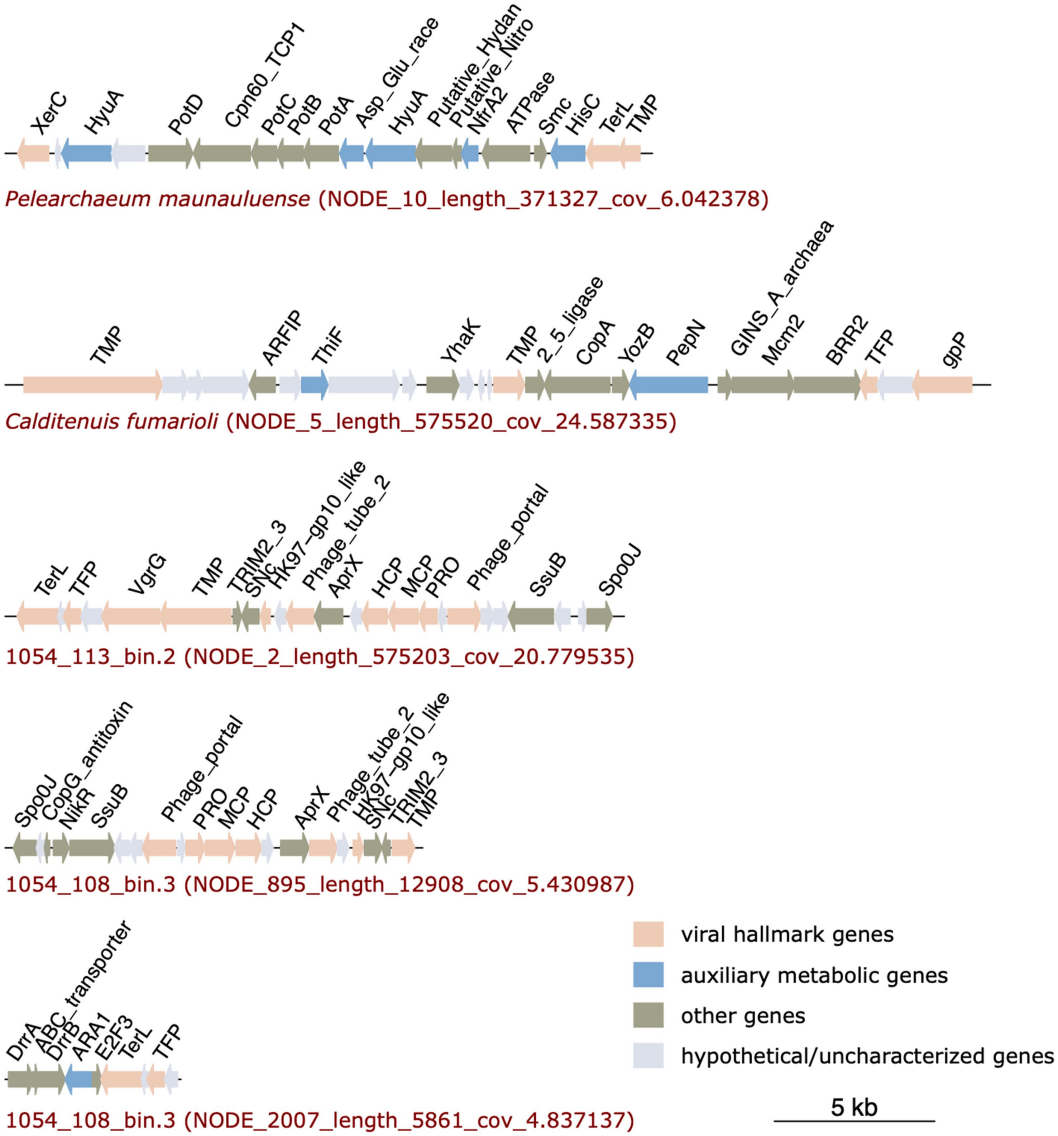
Prophage regions identified in *P. maunauluense*, *C. fumarioli*, and 1054_113_bin.2, and two viral contigs (putative prophage regions) identified in 1054_108_bin.3. The third viral contig of 1054_108_bin.3 is not shown in the figure since it does not include any genes encoding viral hallmark proteins. Full list of the genes and their annotations are shown in Supplementary Table S6.

Viral genes were not detected in the MAG 1054_113_bin.10. However, the MAGs of *P. maunauluense*, *C. fumarioli* and 1054_113_bin.2 include prophage regions, and MAG 1054_108_bin.3 has three viral contigs, which we refer to as putative prophage regions (Figure 4). Although VIBRANT and VirSorter predicted the three contigs of MAG 1054_108_bin.3 to be entirely viral, they very likely belong to prophage regions integrated into the host genome, given MAG 1054_108_bin.3 is a very fragmented and incomplete genome of 167 contigs and 44.34% completeness.

Prophage regions in the MAGs comprise numerous viral hallmark proteins such as the DNA packaging enzyme terminase, phage integrase, a prohead protease involved in phage assembly and maturation, and structural proteins including phage capsid, tail, and portal proteins. In addition to viral hallmark proteins, prophage regions encode proteins associated with viral genome replication such as Staphylococcal nuclease homolog, which is thermostable, replicative DNA helicase Mcm (minichromosome maintenance helicase), RNA 2′,3′-cyclic 3′-phosphodiesterase, and ParB family transcriptional regulator. The replicative DNA helicase Mcm was shown to be frequently recruited from the host as the main replication protein of various crenarchaeal and euryarchaeal mobile genetic elements, including viruses and plasmids (Krupovic et al., 2010, 2019; Kazlauskas et al., 2016). RNA 2′,3′-cyclic 3′-phosphodiesterase was also shown to function as a highly heat-stable cyclic nucleotide phosphodiesterase with GTP-dependent RNA ligase activity in the hyperthermophilic archaeon *Pyrococcus furiosus* (Kanai et al., 2009). The presence of a thermostable nuclease and RNA 2′,3′-cyclic 3′-phosphodiesterase in the provirus regions suggests viruses infecting *Caldarchaeales* might have evolved strategies to replicate their genomes in high-temperature environments. The prophage regions annotated here also carry genes encoding functionally diverse proteins that might contribute to the growth of these archaea in extreme conditions. They are listed in Table 3. These findings are consistent with a previous study reporting that IMGEs found in 20 Thaumarchaeota genomes and one *Caldarchaeales* genome encode multiple AMGs, various dehydrogenases, stress response proteins, membrane transporters of cations and drugs, and chemotaxis protein receiver domains, which could contribute to the fitness, adaptation, and survival of the archaeal hosts by providing mechanisms that respond to stressful environmental conditions, and improve the host metabolic potential (Krupovic et al., 2019).

**Table 3:** Predicted annotations and functional categories of the provirus-encoded genes that might potentially contribute to the host fitness and survival.

Annotation	Functional category
ABC (ATP-Binding Cassette) transporters	Uptake of siderophores, heme, and vitamin B12
Putative ABC type spermidine/putrescine transport system proteins	Quorum sensing
ABC-type multidrug transport system proteins	These proteins yield significant BLAST hits to daunorubicin (an antitumor antibiotic drug) resistance ABC transporter subunits.
Type IV secretory pathway ATPase VirB11/Archaellum biosynthesis ATPase	Cell motility and secretion
Archaeal chaperonin	Heat tolerance
P-type Cu + transporter	Copper resistance
FMN reductase [NAD(P)H]	Auxiliary metabolic gene (AMG) involved in riboflavin (vitamin B2) metabolism
Histidinol-phosphate/aromatic aminotransferase	AMG involved in histidine biosynthesis
N-methylhydantoinase A/oxoprolinase/acetone carboxylase	AMG involved in arginine and proline metabolism
Asp/Glu/Hydantoin racemase	AMG involved in purine metabolism
Aldo/keto reductase	AMG involved in vitamin B6 metabolism
Molybdopterin or thiamine biosynthesis adenylyltransferase	AMG involved in sulfur relay system
Aminopeptidase N	AMG involved in glutathione metabolism
Putative nickel-responsive transcription factor	Regulation of nickel uptake
Putative CopG antitoxin of type II toxin-antitoxin system	Antibiotic resistance, biofilm formation, phage inhibition
Putative phage assembly protein	This protein shares significant sequence identities with phage tail-like nano-machines termed contractile injection systems (CISs) which mediate bacterial cell–cell interactions as either type VI secretion systems or extracellular CISs (Xu et al., 2022).
Subtilisin family serine proteases	These are extracellular peptidases that could play a role in protein remineralization and function at extreme temperatures and pH values.

Viral gene clusters found in the MAGs might belong to Mu-like prophages, as the terminase enzymes encoded in the provirus regions of *P. maunauluense*, 1054_113_bin.2, and 1054_108_bin.3 share significant amino acid identities with Mu phage terminases, and one of the phage tail proteins encoded in the provirus region of *C. fumarioli* shares significant amino acid identities with that of the prophage Mu tail protein. The finding that some viral hallmark proteins in the provirus regions of the MAGs display homology to Mu phage proteins suggests that Mu-like phages could be infecting *Caldarchaeales* hosts. This finding also corroborates a previous study in which Mu-like prophages were found in two *Caldarchaeales* MAGs (Hua et al., 2018). Viruses identified in these MAGs might enhance the metabolic potential of their hosts through horizontal gene transfer events, facilitate niche-specialization, and may reduce competition for resources in extreme habitats such as hot springs and fumaroles found in the study sites presented here. Archaeal viruses are among the most enigmatic viruses known due to a relatively small number of them described to date compared to bacteriophages and eukaryotic viruses and due to their extraordinary genomic diversity and unique morphologies (Prangishvili et al., 2017; Munson-McGee et al., 2018; Wirth and Young, 2020). In this regard, further *in vivo* characterization of the putative *Caldarchaeales* viruses is needed to better understand their ecological roles and elucidate the co-evolution of them with their hosts in extreme habitats.

### CRISPR-Cas systems

**Figure 5:**
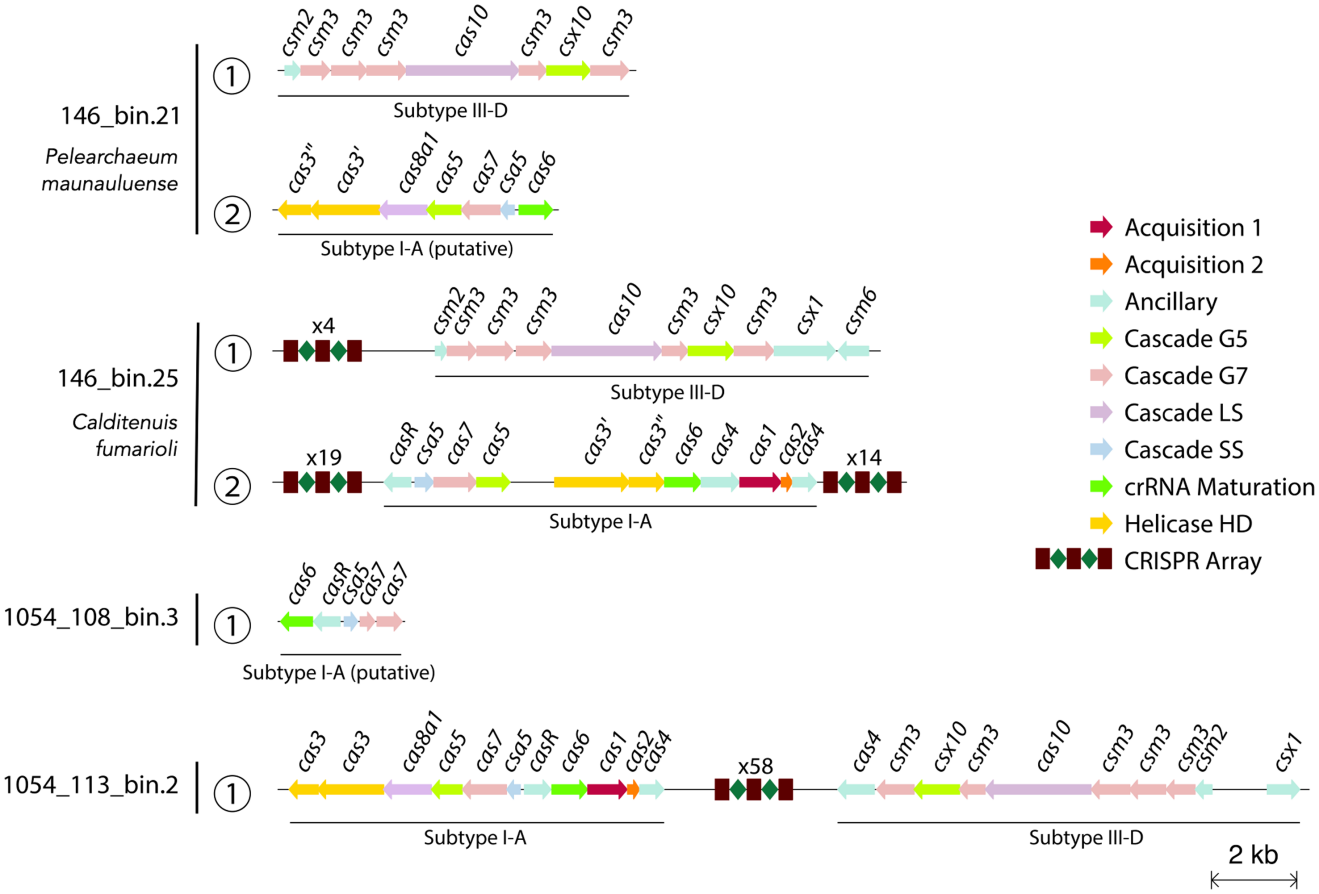
Visualization of predicted CRISPR-Cas arrays and *cas* loci in the contigs of 4 MAGs. Since the MAG 1054_113_bin.10 does not harbor any *cas* loci, CRISPR arrays and *cas* genes identified in 1054_113_bin.10 are not included in the figure. The text above the CRISPR array icon highlights the number of spacers in a CRISPR array and *cas* genes are color-coded according to their functions. The full list of the CRISPR-Cas related genes and arrays identified are shown in Supplementary Tables S8, S9, respectively.

CRISPR-Cas systems in *Caldarchaeales* genomes have been previously reported, with MAGs harboring type-I and type-III CRISPR-Cas systems (Nunoura et al., 2011; Hua et al., 2018). Our analysis shows that type-I and type-III CRISPR-Cas systems might be also present in the MAGs reported here. We found that *P. maunauluense* has two *cas* loci classified as subtype III-D and putative subtype I-A, respectively, in two contigs (Figure 5; Supplementary Table S8). However, neither has a nearby CRISPR array (Supplementary Table S9). In the third contig of *P. maunauluense*, we detected two CRISPR loci, both of which lack *cas* genes in their vicinity. *Calditenuis fumarioli* and 1054_113_bin.2 have both subtype III-D and subtype I-A *cas* loci with nearby CRISPR arrays. 1054_108_bin.3 includes a putative subtype I-A *cas* locus, but this MAG lacks CRISPR arrays, possibly because it is highly incomplete and fragmented. Although we did not recover any *cas* locus in 1054_113_bin 10, we found that it harbors two *cas3* genes in the same contig, which does not contain a CRISPR array. Furthermore, three CRISPR arrays were found in three 1054_113_bin.10 contigs.

### Genes for cell motility and surface attachment

*Pelearchaeum maunauluense*, *C. fumarioli*, and 1054_113_bin.10 encode genes for the following archaeal flagellar components: FlaB, FlaG, FlaH, FlaI, FlaJ, and FlaK (Supplementary Table S3). However, 1054_108_bin.3 only has the gene encoding FlaK. No archaeal flagellar components were identified in 1054_113_bin.2, possibly due to the low level of completeness of this genome. The presence of archaeal flagellar components implies that microbial lineages represented by *P. maunauluense*, *C. fumarioli*, and 1054_113_bin.10 may be motile under certain environmental conditions. In addition, the gene encoding for an archaeal type IV pilin was identified in *P. maunauluense*, *C. fumarioli*, 1054_108_bin.3, and 1054_113_bin.10. *Archaea* type IV pili are involved in biofilm formation, including surface adhesion, microcolony formation, and regulation of flagella-dependent motility (Pohlschroder and Esquivel, 2015). In this respect, biofilm formation may protect against environmental stressors such as low or high pH and toxic chemicals and promote horizontal gene transfer and syntrophy with other microorganisms (van Wolferen et al., 2018). Given the role of type IV pili in archaeal biofilm formation, organisms from which *P. maunauluense*, *C. fumarioli*, 1054_108_bin.3, and 1054_113_bin.10 were derived may be able to form biofilms. Moreover, two chemotaxis-related genes (methyl-accepting chemotaxis protein and a heme-based aerotactic transducer) were found in *P. maunauluense* genome. Taken together, motility genes in *P. maunauluense*, *C. fumarioli*, 1054_108_bin.3, and 1054_113_bin.10, and chemotaxis genes in *P. maunauluense*, provide genomic evidence that these lineages might be able to move toward favorable environments within these geothermal habitats.

### Pangenomic analysis of three distinct *Caldarchaeales* clades

**Figure 6:**
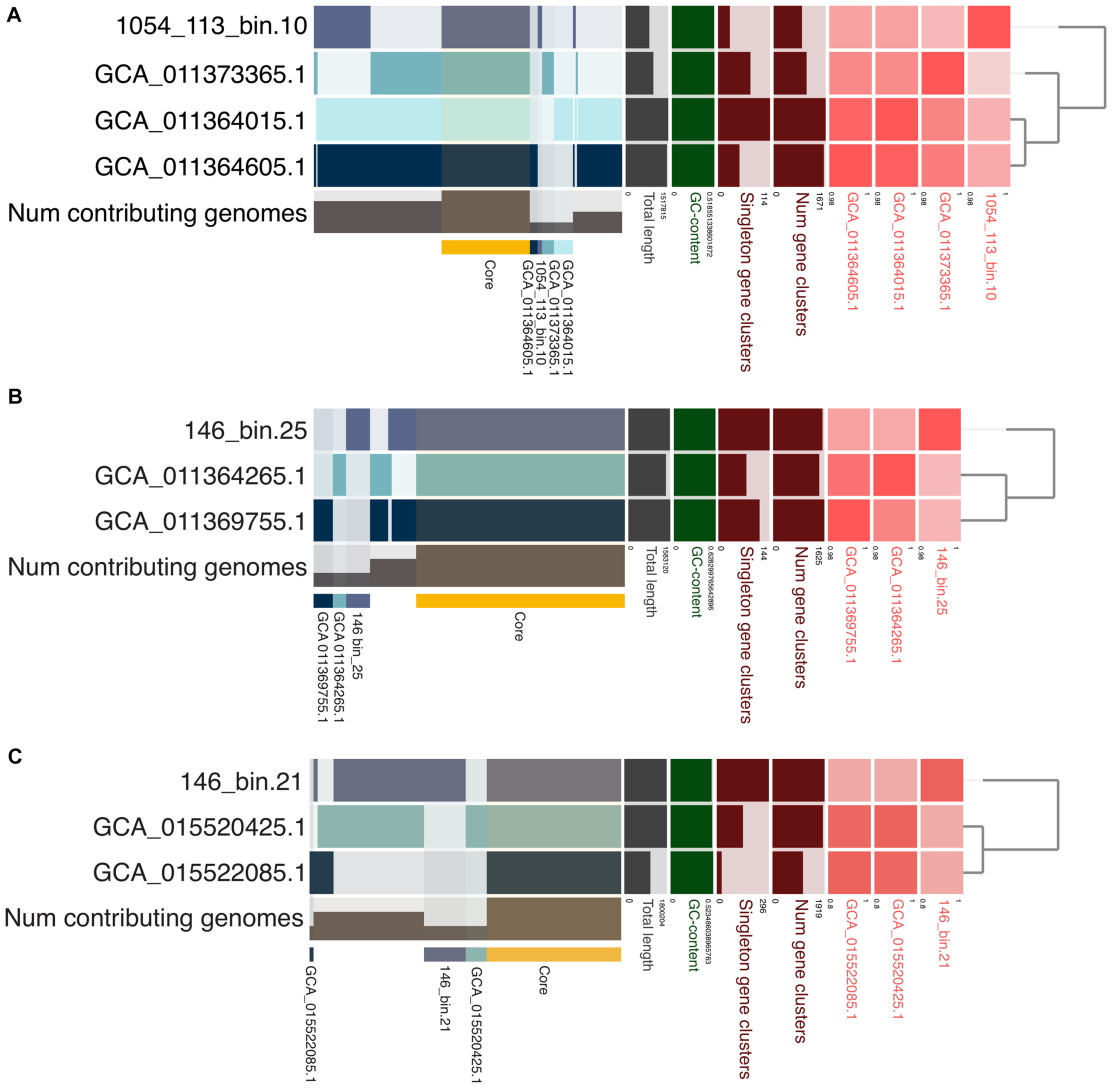
Pangenomic analysis of **(A)** 1054_113_bin.10 from Chile, **(B)** 146_bin.25 (*C. fumarioli*) from Hawai’i, and **(C)** 146_bin.21 (*P. maunauluense*) from Hawai’i. Phylogenomic trees show the relationship between the MAGs compared. The red boxes in various shades represent average nucleotide identities (ANI) between the MAGs, with the darkest shade indicating highest ANI values. Core gene clusters found in all MAGs in each comparison are highlighted with a yellow horizontal bar. Unique gene clusters present in respective MAGs are also highlighted similarly using different colors and labeled with their respective accession numbers or bin names.

The pangenomic comparison of 1054_113_bin.10 with its closest relatives from GTDB showed 1,844 genes in this pangenome, 528 of which are core, 26 which are unique, and that 19 assigned to the bin have COG categories (Figure 6A; Supplementary Table S11). Most of these gene clusters belong to “Amino acid transport and metabolism,” and “Inorganic ion transport and metabolism” COG categories that comprise ABC-type nitrate/sulfonate/bicarbonate transport system components, and ABC-type dipeptide/oligopeptide/nickel transport system components. There are also two gene clusters of the COG category “Defense mechanisms” that encode toxin-antitoxin (TA) system-associated proteins. Two reference genomes from Jinze hot spring sediment environment, China (GCA_011364605.1 and GCA_011364015.1), have 265 gene clusters that were not found in 1054_113_bin.10 or the reference genome (GCA_011373365.1) from Gongxiaoshe hot spring sediment, China. Most of the 173 gene clusters assigned to COG categories belong to “Energy production and conversion,” “Amino acid transport and metabolism,” and “Defense mechanisms.” The gene clusters of “Energy production and conversion” mainly comprise genes involved in the non-phosphorylated Entner-Doudoroff pathway, TCA cycle, and pyruvate oxidation. Gene clusters of “Defense mechanisms” mostly include genes associated with toxin-antitoxin (TA) systems, CRISPR-Cas systems, and ABC-type multidrug transport systems. On the other hand, COG category “Inorganic ion transport and metabolism” contain gene clusters encoding sulfite exporters, an ABC-type Fe^3+^-hydroxamate transport system component, ABC-type cobalamin/Fe^3+^-siderophores transport system components, a phosphate uptake regulator, and predicted Fe^2+^/Mn^2+^ transporter. There is also one gene cluster of the COG category “Mobilome: prophages, transposons” that encodes Mu-like prophage FluMu protein gp28, indicating the occurrence of past viral infections. The reference genome (GCA_011373365.1) from China’s Gongxiaoshe hot spring sediment contains 71 unique gene clusters, 34 of which are annotated; most of the annotated gene clusters belong in the COG categories “Carbohydrate transport and metabolism,” “Coenzyme transport and metabolism,” and “Post-translational modification, protein turnover, chaperones” that includes gene clusters encoding chaperonin GroEL.

The pangenomic analysis of *C. fumarioli* and its closest relatives from GTDB revealed 1869 gene clusters in the pangenome, with 1,253 core gene clusters, and 146 gene clusters unique to *C. fumarioli*, 68 of which were annotated to COG categories (Figure 6B; Supplementary Table S12). The COG categories “Carbohydrate transport and metabolism” and “Defense mechanisms” contained the largest number of unique gene clusters. The “Carbohydrate transport and metabolism” category includes ABC-type sugar transport system components and a 5-Carboxyvanillate decarboxylase (LigW) that catalyzes the conversion of 5-carboxyvanillate to vanillate in the biochemical pathway for the degradation of lignin (Vladimirova et al., 2016). The “Defense mechanisms” category mainly includes CRISPR-Cas system-associated proteins, and additionally a few toxin/antitoxin (TA) system proteins, and an ATPase component of the ABC-type multidrug transport system. Considering that two reference genomes used in the pangenome analysis of *C. fumarioli* were obtained from a hot spring sediment metagenome and they belong to the same species as *C. fumarioli*, and the proteins associated with CRISPR-Cas and TA systems are among the most common unique gene clusters of *C. fumarioli*, viruses infecting *Archaea* may be more prevalent in fumaroles than in hot springs, and the unique gene clusters might reflect adaptation strategies to the fumarole environment of organisms represented by *C. fumarioli*.

For example, one of the unique gene clusters in the COG category “Energy production and conversion” encodes nitrite reductase (NO-forming), part of denitrification, and an “Intracellular trafficking, secretion, and vesicular transport” category unique gene cluster that encodes VirB4 ATPase of the Type IV secretion systems, which mediate the transfer of proteins and DNA across the bacterial cell membrane and play important roles in bacterial pathogenesis and horizontal transfer of antibiotic resistance (Walldén et al., 2012). Moreover, the presence of “Inorganic ion transport and metabolism” category unique gene clusters comprising components of ABC-type Fe^3+^ transport system, ABC-type nitrate/sulfonate/bicarbonate transport system, sulfite exporter TauE/SafE/YfcA and related permeases, and enzymes associated with assimilatory sulfate and assimilatory nitrate reductions, might provide insights into the adaptations of *Caldarchaeales* to the physicochemical characteristics of the fumaroles. Conversely, two reference genomes from the Jinze hot spring sediment share 110 gene clusters that were also not found in *C. fumarioli*. Among these, 54 were annotated, and a large number belong in the COG categories “Amino acid transport and metabolism,” “Translation, ribosomal structure and biogenesis,” “Nucleotide transport and metabolism,” and “Replication, recombination and repair.” One gene cluster in the COG category, “Inorganic ion transport and metabolism,” was annotated as a predicted copper/silver-translocating P-type ATPase, which suggests potential involvement in heavy metal stress resistance. However, none of these 110 gene clusters fell into the “Defense mechanisms” category, including CRISPR-Cas and toxin/antitoxin systems.

With respect to the pangenome of *P. maunauluense* and its closest GTDB relatives, the total number of gene clusters was 2,209, with 953 core gene clusters, and 296 gene clusters unique to *P. maunauluense* is 296. Of the latter, 173 were not assigned to any COG category (Figure 6C; Supplementary Table S13). Gene clusters assigned to the COG category “Defense mechanisms” comprise the majority among 123 annotated unique gene clusters. These “Defense mechanism” gene clusters consist of proteins associated with CRISPR-Cas and toxin-antitoxin systems (TAs). TAs are small genetic modules found on bacterial mobile genetic scaffolds such as plasmids, as well as on bacterial and archaeal chromosomes (Yamaguchi et al., 2011; Yang and Walsh, 2017; Song and Wood, 2020). They typically consist of two elements: a toxin that inhibits an essential cellular process, and an antitoxin that counteracts its cognate toxin (Jurėnas et al., 2022). TAs play a critical role in the distribution and evolution of bacterial antibiotic resistance (Yang and Walsh, 2017) and promote cell survival in their native habitat (Page and Peti, 2016; Yang and Walsh, 2017). It has also been demonstrated that they are involved in multi-resistant plasmid maintenance, stress management, and biofilm formation in *Bacteria* (Yang and Walsh, 2017).

Song and Wood (2020) reported that a primary physiological function of chromosomally encoded TA systems in bacteria is phage inhibition, that some CRISPR-Cas system elements are derived from TA systems, and some CRISPR-Cas systems mimic TA systems by reducing host metabolism to inhibit phage propagation. Given a substantial number of annotated unique gene clusters of *P. maunauluense* belong in CRISPR-Cas and TA systems, viruses of *Archaea* might be relatively more abundant in the fumarole environment from which *P. maunauluense* was recovered than in the marine hydrothermal vent from which two MAGs from the same genus were obtained. Other unique gene clusters of *P. maunauluense* comprise COG categories “Mobilome: prophages, transposons,” which includes transposases, “Inorganic ion transport and metabolism,” which includes a high affinity permease of iron and lead ions, and superoxide dismutases implicated in oxidative stress resistance, “Nucleotide transport and metabolism,” “Carbohydrate transport and metabolism,” “Amino acid transport and metabolism,” “Coenzyme transport and metabolism,” “Lipid transport and metabolism,” “Secondary metabolites biosynthesis, transport and catabolism,” “Cell motility,” “Cell wall/membrane/envelope biogenesis,” and “Energy production and conversion” which includes tungsten-containing aldehyde:ferredoxin oxidoreductases and tetrathionate reductase subunit A, “Post-translational modification, protein turnover, chaperones” that includes predicted dithiol-disulfide isomerases which might be involved in oxidative stress tolerance (Khairnar et al., 2013), “Replication, recombination and repair” that includes DNA repair photolyase, “Signal transduction mechanisms,” “Transcription,” and “Translation, ribosomal structure and biogenesis.”

On the other hand, 113 gene clusters belong exclusively to the two MAGs from the marine hydrothermal vent and were not found in *P. maunauluense*. Among them, 66 were assigned to COG categories. Most of the gene clusters belong to the COG categories, “Energy production and conversion,” “Amino acid transport and metabolism,” “Lipid transport and metabolism,” and “Defense mechanisms,” with the first one containing the largest number of gene clusters. “Energy production and conversion” includes perchlorate reductase subunit alpha, which catalyzes the reduction of perchlorate to chlorite, and allows anaerobic growth on perchlorate as the electron acceptor; also included is tetrathionate reductase subunit B, a tungsten-containing aldehyde:ferredoxin oxidoreductase, and heterodisulfide reductase, subunit A (polyferredoxin)/coenzyme F420-reducing hydrogenase, delta subunit complex that may be involved in flavin-based electron bifurcation. There is also one gene cluster annotated as “Phage portal protein BeeE,” which is a member of the COG category “Mobilome: prophages, transposons,” implying past viral invasions. Interestingly, the “Defense mechanisms” category does not include CRISPR-Cas system-associated proteins, but it does include TA system proteins, an antibiotic ABC transporter ATP-binding protein, and an enamine deaminase RidA (reactive intermediate deaminase A) that protects metabolic enzymes against damage by reactive intermediates (Irons et al., 2020). Some other gene clusters that might contribute to the survival of *Caldarchaeales* in hydrothermal vent habitats encode manganese-containing antioxidant catalase (includes spore coat protein CotJC) in the COG category “Inorganic ion transport and metabolism,” and an activator of 2-hydroxyglutaryl-CoA dehydratase (involved in the amino acid fermentation pathway) which were assigned to “Lipid transport and metabolism,” plus a subtilisin family serine protease which could display hyperthermostable features, in the “Post-translational modification, protein turnover, chaperones” category.

We should also note that even though these pangenomic analyses could offer a perspective on organism- and niche-specific features and adaptations in *Caldarchaeales*, it is important to consider the incompleteness of the MAGs reported here, and of the reference genomes included in the analyses when interpreting the results. Due to the incomplete nature of many closely related MAGs in publicly available databases, we were only able to include relatively small numbers of their genomes in the pangenomic analyses. It is also important to note that the third pangenomic analysis of *P. maunauluense* MAG involved comparisons with genomes from at least two or more different species whereas the other two pangenomic included within-species level genomes. As such, a much larger number of core gene clusters may have been identified in the multi-species pangenomic analysis.

## Conclusion

Metabolic reconstruction of five MAGs based on their gene content revealed that these members of the *Archaea* order *Caldarchaeales* display metabolic flexibility. They are equipped to utilize both organic substrates (i.e., glucose, amino acids/peptides, and lipids) and inorganic substrates (i.e., nitrite, hydrogen sulfide, and carbon monoxide) as electron donors, indicating a mixotrophic lifestyle.

Significantly, the fact that some MAGs encode enzymes involved in the dissimilatory tetrathionate reduction, dissimilatory reduction of nitrate and nitrite, and acetate fermentation in addition to the components of the oxidative phosphorylation complex IV attests these *Caldarchaeales* members may thrive in aerobic, microaerophilic, and anoxic environments. The presence of genes involved in oxidative stress tolerance, plus that encoding pyruvate ferredoxin/flavodoxin oxidoreductase, which enables acetyl-CoA production under anoxic conditions, and the gene encoding pyruvate dehydrogenase, which enables acetyl-CoA production under oxic conditions, suggest that these archaea have adapted to fluctuating oxygen concentrations in their habitat. Moreover, all MAGs possess the metabolic potential to fix carbon dioxide through the roTCA cycle. Also, *Caldarchaeales* members in Hawai‘i might be capable of carrying out carbon dioxide fixation via the 3HP/4HB or DC/4HB cycle based on the occurrence of some key marker genes in the MAGs, although these pathways are not complete in any of the MAGs. Taken together, the wide range of metabolic capabilities displayed by these archaea might render them selectively advantageous, in case they encounter various extreme environmental conditions.

The existence of proviral regions in the MAGs indicates that these *Caldarchaeales* members might have undergone viral infections. Furthermore, the fact that all the MAGs encode Cas proteins and CRISPR arrays suggests that they developed a response mechanism against viral stress. Intriguingly, proviral regions in the MAGs were determined to encode AMGs, different membrane transporters, ABC-type spermidine/putrescine transport system proteins implicated in quorum sensing, genes playing a role in cell secretion, and genes involved in resistance to antibiotics, heavy metals, and extreme temperature and pH values along with the viral signature genes. These findings corroborate previous studies reporting that prophage regions in microbial host genomes might comprise genes with functions which contribute to the host fitness and adaptation in myriad habitats. Lastly, increasing the number of *Caldarchaeales* representative genomes through this work has improved the phylogenomic resolution of this group of enigmatic archaea and supports their status as a clade distinct from the Thaumarchaeota. Many gaps remain in our understanding of *Caldarchaeales* biology in geothermal habitats, but the genomic information gleaned here may help us design conditions for their growth and physiological characterization in the laboratory.

## Data availability statement

The original contributions presented in the study are publicly available. This data can be found at: https://www.ncbi.nlm.nih.gov/bioproject/?term=PRJNA872141.

## Author contributions

CC and DL led the BASALT project to collect samples from Mauna Ulu. SC collected the samples from Chile. AD provided funding for metagenomic sequencing. RP, PC, and SD coordinated sample processing and obtained metagenome sequencing. MS performed metagenome assembly and binning, and identification of *Caldarchaeales* in public databases. MB performed metabolic pathway analysis, and analysis of viral and CRISPR regions. MS and MB performed pangenomic analyses. JS conducted taxonomic classifications and phylogenomic analyses and created the metabolic pathway overview figure. MB, MS, and JS prepared the initial draft of the manuscript. All authors contributed to the article and approved the submitted version.

## Funding

This research was supported by NASA Exobiology grant (80NSSC18K1064) to AD, PC, and SD; a NASA Astrobiology Institute (NAI)-CAN7 grant (13-13NAI7_2-0018) to SC; a Science and Technology Facilities Council (STFC) grant no. ST/V000586/1 to CC; CCAS startup and University Facilitating (UFF) funds from The George Washington University to JS; and the U.S. Department of Energy, Office of Science, Biological and Environmental Research Division, under award number LANLF59G to PC.

## Conflict of interest

The authors declare that the research was conducted in the absence of any commercial or financial relationships that could be construed as a potential conflict of interest.

## Publisher’s note

All claims expressed in this article are solely those of the authors and do not necessarily represent those of their affiliated organizations, or those of the publisher, the editors and the reviewers. Any product that may be evaluated in this article, or claim that may be made by its manufacturer, is not guaranteed or endorsed by the publisher.

## Footnotes

^1^ https://github.com/tseemann/barrnap

^2^ vogdb.org

^3^ https://github.com/tseemann/barrnap
