# Supplementary material for: Metabolic versatility of Caldarchaeales from geothermal features of Hawai’i and Chile as revealed by five metagenome-assembled genomes
Source: Front Microbiol. 2023 Sep 20;14:1216591. doi: 10.3389/fmicb.2023.1216591 (PMC10547907; doi:10.3389/fmicb.2023.1216591)
Supplement: Supplementary file 14 [file Data_Sheet_1.docx]

Supplementary Material

Insights from five metagenome-assembled genomes of *Caldarchaeales* from terrestrial geothermal features of Hawai‘i and Chile

Manolya Gul Balbay^1,#^, Maximillian D. Shlafstein^1,#^, Charles Cockell^2^, Sherry L. Cady^3^, Rebecca D. Prescott^,2,6,7,8†*^, Darlene S. S. Lim^4^, Patrick S. G. Chain^5^, Stuart P. Donachie^6^, Alan W. Decho^7^, Jimmy H. Saw^1*^

^1^ Department of Biological Sciences, The George Washington University, Washington, DC, USA

^2^ UK Centre for Astrobiology, University of Edinburgh, Edinburgh, UK

^3^ Department of Geology, Portland State University, Portland, OR, USA

^4^ NASA Ames Research Center, Moffett Field, CA, USA

^5^ Los Alamos National Laboratory, Los Alamos, NM, USA

^6^ School of Life Sciences, University of Hawai‘i at Mānoa, Honolulu, HI, USA

^7^ Department of Environmental Health Sciences, University of South Carolina, Columbia, SC, USA

^8^ Department of Biology, University of Mississippi, Oxford, MS, USA

^#^ These authors contributed equally.

^†^ Current address: Department of Biology, University of Mississippi, Oxford, MS, USA

* Corresponding authors

Correspondence email address: jsaw@gwu.edu

# Supplementary Data

**Supplementary Data File 1.** Phylogenomic tree produced by IQ-Tree program showing all archaea included in the inference.

**Supplementary Data File 2.** Prodigal-annotated protein-coding genes identified in the five MAGs presented in this study.

**Supplementary Data File 3.** Raw output files produced by PSORTb web tool to predict subcellular localization of MEROPS hits identified by the METABOLIC tool.

# Supplementary Tables

**Supplementary Table 1.** List of environmental features and samples obtained from NCBI SRA databases that showed highest relative 16S rRNA gene abundances of Aigarchaeota.

**Supplementary Table 2.** Overview of major metabolic pathways deemed to be present or absent in the five MAGs based on pathway reconstruction analyses.

**Supplementary Table 3.** Detailed list of major metabolic pathways and key enzymes identified in the five MAGs. The second column lists their KEGG ortholog IDs and the third column lists the products the genes encode. Number of copies of the genes present are also listed in subsequent columns under respective MAGs.

**Supplementary Table 4.** Ghostkoala annotations of all five MAGs.

**Supplementary Table 5.** eggNOG mapper annotations of all five MAGs.

**Supplementary Table 6.** Detailed annotation of viral and auxiliary metabolic genes identified in four of the MAGs.

**Supplementary Table 7.** Transposases identified in some of the MAGs.

**Supplementary Table 8.** Genes involved in CRISPR-Cas systems along with their classifications and gene coordinates in the MAGs.

**Supplementary Table 9.** CRISPR arrays and their coordinates identified in some of the MAGs.

**Supplementary Table 10.** Output from METABOLIC tool.

**Supplementary Table 11.** Gene clusters identified in the Anvi’o pangenomic comparison of 1054_113_bin.10 with genomes of closely related organisms.

**Supplementary Table 12.** Gene clusters identified in the Anvi’o pangenomic comparison of 146_bin.25 with genomes of closely related organisms.

**Supplementary Table 13.** Gene clusters identified in the Anvi’o pangenomic comparison of 146_bin.21 with genomes of closely related organisms.

# Supplementary Figure(s)

**Supplementary Figure S1.** Dr. Sherry Cady collects biofilm samples at El Tatio’s Poppy Pool along the high temperature surge zone in November 2018.
