# Supplementary figures and images for: Metabolic versatility of Caldarchaeales from geothermal features of Hawai’i and Chile as revealed by five metagenome-assembled genomes
Source: Front Microbiol. 2023 Sep 20;14:1216591. doi: 10.3389/fmicb.2023.1216591 (PMC10547907; doi:10.3389/fmicb.2023.1216591)

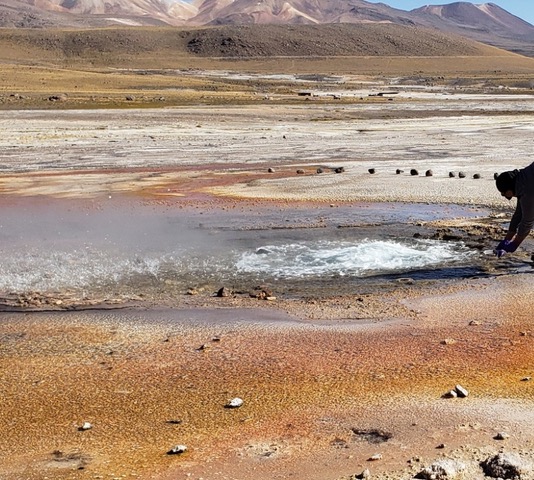

Supplement: Supplementary file 18 [file Image_1.jpeg]
